# What can attribution methods show us about chemical language models?†‡

**DOI:** 10.1039/d4dd00084f

**Published:** 2024-07-18

**Authors:** Stefan Hödl, Tal Kachman, Yoram Bachrach, Wilhelm T. S. Huck, William E. Robinson

**Affiliations:** a Physical Organic Chemistry, Radboud University Heyendaalseweg 135 6525AJ Nijmegen The Netherlands william.robinson@ru.nl; b Artificial Intelligence, Donders Institute, Radboud University Thomas Van Aquinostraat 4 6525GD Nijmegen The Netherlands; c Google Deepmind London UK

## Abstract

Language models trained on molecular string representations have shown strong performance in predictive and generative tasks. However, practical applications require not only making accurate predictions, but also explainability – the ability to explain the reasons and rationale behind the predictions. In this work, we explore explainability for a chemical language model by adapting a transformer-specific and a model-agnostic input attribution technique. We fine-tune a pretrained model to predict aqueous solubility, compare training and architecture variants, and evaluate visualizations of attributed relevance. The model-agnostic SHAP technique provides sensible attributions, highlighting the positive influence of individual electronegative atoms, but does not explain the model in terms of functional groups or explain how the model represents molecular strings internally to make predictions. In contrast, the adapted transformer-specific explainability technique produces sparse attributions, which cannot be directly attributed to functional groups relevant to solubility. Instead, the attributions are more characteristic of how the model maps molecular strings to its latent space, which seems to represent features relevant to molecular similarity rather than functional groups. These findings provide insight into the representations underpinning chemical language models, which we propose may be leveraged for the design of informative chemical spaces for training more accurate, advanced and explainable models.

## Abstract 2

Chemical language models can be fine-tuned to predict properties, like water solubility, given molecular inputs. This work investigates how these predictions may be explained.

## Graphical abstract

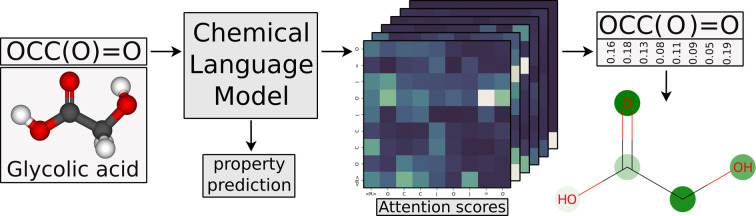

## 1 Introduction

Chemical structures encode the physicochemical properties of compounds and their interactions. However, creating useful computational representations of chemical structures that can be regressed onto these properties remains a significant challenge. Predictive models based on molecular fingerprints have been in use for decades,^1,2^ but the design of functional molecular systems largely remains an experimental science. This situation is in part due to the challenge of building models that can generalise to predict the properties of compounds outside of the chemical space of their training data. Chemists broadly approach the problem of generalisation by searching for molecular features that correlate with properties, a concept formalised in quantitative structure–property relationship modelling (QSPR).^3^ In such models, interpretability is key, and features are often specifically “hand selected” with guidance from expert chemical knowledge. Thus, these approaches rely upon in-depth knowledge of a chemical domain and property landscape for efficient model development and require extensive (re-)design when chemical components and target properties change.

Recent advances in deep learning have led to the proliferation of the transformer architecture^4^ across many domains, achieving state-of-the-art results in vision,^5^ language^6^ and life sciences.^7^ These models are pretrained in a “self-supervised” fashion to learn expressive representations of their training data, which can then be used for predictive or generative tasks. In the domain of chemistry, transformers trained on large databases of SMILES strings^8^ are a new class of models called Chemical Language Models (CLMs).^9^ Such CLMs have achieved state-of-the-art results in property,^10^ reaction and retrosynthesis^10,11^ prediction, establishing the potential of these generative models as tools for the design of molecular systems with tailored properties.^12–15^

Focusing on property prediction, aqueous solubility has been explored extensively due to the availability of large datasets of experimental measurements.^16–18^ The SolProp^18^ data collection contains the largest currently available dataset for aqueous solubility with ∼11.8 K experimental measurements. Transformer-based architectures have been explored to directly predict aqueous solubility, and among them is SolTranNet,^19^ which adapts the MoleculeAttentionTransformer^20^ architecture for the AqSolDB^17^ dataset. Another recent study pretrains a SMILES language model from scratch, focusing on directly predicting the solvation free energy as well as solubility in organic solvents.^21^

In the context of QSPR, transformers are particularly compelling due to their ability to encode complex information about molecular structure in a compressed latent representation, which may be regressed onto a property. However, this ability comes at the cost of explainability. Latent representations learned by transformers are poorly understood, particularly in comparison to more conventional, expert-designed QSPR methods. Thus, the time-tested chemists' strategy of generalisation *via* the mapping of molecular features to properties is significantly hindered in the case of using complex model architectures such as transformers.

Explainable AI (XAI) techniques aim to explain the predictions of deep learning models, ranging from approaches such as saliency maps^22^ and model-agnostic techniques such as SHAP^23^ to more specialised, domain-specific techniques.^24,25^ Recent work in the domain of natural language processing has attempted to explore the inner workings of transformers,^26,27^ but it is not clear whether current explainability techniques are effective in explaining the predictions of CLMs. Adapting transformer architectures to varying domains requires modifications and adjustments to account for differences in representations and tasks, and the same is true for explainability techniques. Gradient-based attribution methods constitute a class of XAI techniques that are used to quantify the influence of each input feature on the prediction.^28–31^ Attribution methods are particularly suited to chemistry as feature influence can be easily mapped to molecular structures and visualised. Visualisation allows for a familiar and intuitive inspection of an explanation of a model, which chemists can use for both evaluation and gaining insight into model predictions.

In this work, we explore the application of attribution methods to a CLM. We use the pretrained encoder of MegaMolBART^32^ to generate latent representations based on SMILES^8^ strings. These molecular representations are regressed onto aqueous solubility values using experimental aqueous solubility measurements from the SolProp database^18^ to train a model that predicts solubility using molecular structures as encoded in SMILES strings. We then explore the application of attribution methods (a transformer-specific attribution technique^33,34^ and SHAP^23^) towards explaining the solubility predictions of MegaMolBART. The explanations of these methods are then compared with those of models using extended connectivity fingerprints as an established molecular representation method. We find that both explainability techniques for the CLM produce sparse attributions focusing on tokens, but neither technique can fully explain the model's solubility predictions due to distinct inherent limitations in both techniques.

## 2 Methods

### 2.1 Data

The AqueousSolu dataset from the SolProp data collection^18^ (Version v1.2, July 1, 2022) was used for all experiments, which contains 11 804 experimental log(*S*) measurements of aqueous solubility at 298 K. We split the dataset into a train, validation and test sets using random, “accurate”^18^ and scaffold^35^ split strategies. For the “random” test setting, a 10% test set obtained from a fixed random seed is set aside. The remaining molecules are repeatedly split randomly into a 90% training and a 10% validation set three times for cross validation, using the same splits for all models. The SolProp authors propose and evaluate an “accurate” test set with low experimental uncertainty, which is obtained by selecting all molecules with more than 1 measurement, where the standard deviation does not exceed 0.2. In this case, the random seed only affects the random sampling for the five splits into train and validation sets for cross validation.

### 2.2 Extended connectivity fingerprints

We used the established extended connectivity fingerprints (ECFPs) as a predictive and interpretable baseline model. ECFP bits signify the presence or absence of particular molecular substructures, which are not defined beforehand and can thus be viewed as a generalisation of predictive approaches using handpicked substructures like Crippen's log(*P*).^1^ We focussed on ECFPs since they are also interpretable by visualizing the substructures that caused a particular bit to be activated and quantifying how much the substructure contributed to the prediction from the corresponding weight coefficient in the regression head. We generated binary feature vectors with 512 or 2048 bits and radius 2 using RDKit (version 2023.3.3)^36^ and converted them to numpy arrays as input for the regression head. Specifically, we used the “AllChem.GetMorganFingerprintAsBitVect” function, which enables the reconstruction of each bit's substructures using the “GetOnBits” and “FindAtomEnvironmentOfRadiusN” functions, which is necessary to attribute regression coefficients towards all constituent atoms of the substructure.

### 2.3 Pretrained transformers

In recent years, the deep learning approach forgoing handcrafted fingerprints in favor of a learnable latent vector has achieved state-of-the-art performance.^10,32,37,38^ In this work, we focus on using SMILES strings as source molecular representations. Other molecular line notations, such as SELFIES,^39^ are available. However, we have not investigated them here. Previous work on training the ChemBERTa model has noted that there was no significant difference in model performance between using SMILES and SELFIES as molecular language input.^38^ Furthermore, no pretrained CLMs for SELFIES are available at comparable quality and scale compared to those available for SMILES.

Rather than converting SMILES strings into another representation, chemical language models are transformers that operate directly on string-based representations like SMILES. The transformer applied in this work consists of two separate stacks of layers, respectively, the encoder and the decoder. After tokenizing the input string with the tokenizer, the encoder maps these *T* tokens to a latent representation of the same length, usually of dimensionality *T* × 512. Only this latent representation is necessary for property prediction, but the decoder is needed during pretraining to learn to construct this expressive latent representation in the first place. During pretraining, the decoder aims to reconstruct the original input tokens after passing through the encoder, which can be viewed as an information bottleneck where the model has to leverage a compressed representation to minimize the reconstruction error. In addition to this information bottleneck, the input is randomly corrupted through masking or noise, which further increases the difficulty and helps to obtain features that generalise well. BERT^40^ and BART^41^ style models mask out random subsets of the input tokens, forcing the model to learn to reconstruct the inputs. This lets the model learn representations that can differentiate between the data points and produce outputs that are probable given the distribution of the pretraining dataset, effectively learning to represent chemical space. The CLM thus needs to learn to encode a representation of molecular structures directly from molecular strings, which makes it suitable for both predictive and generative tasks. The advantage of using learned latent representations for property prediction stems from the scale of unlabeled data these models are able to leverage during pretraining.^42^

### 2.4 MegaMolBART

In this work, we selected MegaMolBART as a pretrained CLM. The code and pretrained weights for this model are readily available and open source, fulfilling several criteria that are useful for our investigations into using CLMs for property prediction. MegaMolBART is based on the Chemformer^10^ architecture, a “self-supervised” transformer model that uses SMILES strings as input. Using SMILES as a molecular representation is beneficial, as it is compact in comparison to a full molecular graph representation and is readily available in databases. SELFIES^39^ is another popular line notation that could be considered as input to the CLM. A model pretrained on ∼2 million SELFIES strings is available.^43^ As mentioned above, work on developing ChemBERTa suggests that there is no benefit of using SELFIES over SMILES as chemical language input.^38^ Furthermore, MegaMolBART was trained on ∼1.45 billion SMILES strings,^32^ while Chemformer was trained on ∼100 million.^10^ It has been shown that training LLMs on larger data sets leads to higher performance.^42,44^ Thus, we expect the SMILES-based MegaMolBART model to be the most powerful CLM available at this time.

The MegaMolBART model was obtained through the provided “docker” container image (version v0.2:0.2.0) from the Github repository.^32^ The pretrained PyTorch model and weights were accessed directly to avoid the latency of the “InferenceWrapper” and enable fine-tuning of the encoder. The “RegExTokenizer” vocabulary was extended with a 〈R〉 token, and thus the “tokenize” function was adjusted to prepend the 〈R〉 token. The “encode” function of MegaMolBART takes the SMILES string and produces latent representations of dimensionality (*T* × 512), which include *T* tokens of the SMILES string and 〈PAD〉 tokens to pad up to the maximum token size of the batch. We applied two approaches to reduce this variable-length matrix into a 1 × 512 dimensional vector suitable for the regression head. The “average-pooling” (avg) approach used by MegaMolBART reduces the encoded representation by pooling a tokenwise average of the matrix. Alternatively, we prepend a “readout” token (〈R〉) to the tokenized SMILES string, which aggregates information throughout the encoder and is used to explain the model by attributing relevance from the prediction using this 〈R〉 token (see XAI methods).

### 2.5 Regression methods

Latent molecular representations were regressed onto scalar solubility values using linear (lin, ([512 × 1])) or hierarchical regression heads (hier, three sequential projections of size [512 × 64] → [64 × 64] → [64 × 1] with interleaved ReLU nonlinearities). We applied LayerNormalization for both regression heads before the first projection layer, and for the linear variant we did not use a bias term. Regression tasks were performed using either the pretrained (hereby referred to as ‘frozen’) model or fine-tuning (-ft) the MegaMolBART encoder. During training of the frozen MegaMolBART model, only the weights in the regression head were trained. Fine-tuning was performed by training both the regression head and the ∼20 M parameters of the encoder. For ECFP fingerprints, we additionally fit two established machine learning models,^45^ specifically a support vector regressor (SVR) with the “radial basis function” kernel and random forest regressor (RF) using scikit-learn.^46^

### 2.6 Computational resources

All MegaMolBART models and ECFPs with regression heads were trained on a single Nvidia RTX 2080 GPU using PyTorch and PyTorch Lightning's “Trainer” for 30 epochs with “precision = 16”, which allows for a maximum batch size of 48 due to GPU memory constraints (8 GB). We used the PyTorch AdamW optimizer with betas 0.9 and 0.999, and the learning rate was dynamically adjusted by PyTorch Lightning's “trainer” function “auto_lr_find”. The HuberLoss loss criterion was chosen to balance the mean absolute error (MAE) and root mean squared error (RMSE). One 30-epoch fine-tuning run of MegaMolBART took ∼7 minutes, and the full pipeline for one MegaMolBART model variant took ∼40 minutes depending on the model variant, test set split, number of cross-validation splits and explainability technique.

### 2.7 XAI methods

#### 〈R〉 token

Since the number of tokens and thus the number of latent vectors are of variable length, a readout function is necessary to obtain a single vector of static shape. The average as the readout function is unsuitable in the context of explainability as it hinders the attribution of importance through the backpropagation of the prediction to the input string, since the individual contribution of each token is uniformly distributed during backpropagation through the average-pooling step. We avoided this pooling step by prepending a “readout” token 〈R〉 to the tokenized SMILES string, which aggregates information throughout the encoder. Only the feature vector of this 〈R〉 token is processed in the regression head, which allows us to obtain the relevance of all other tokens to the 〈R〉 token from the pairwise attention weights and backpropagate gradients from the regression prediction to the input tokens. The vocabulary of the MegaMolBART tokenizer thus needs to be extended with a 〈R〉 token, which corresponds to the regression equivalent of the 〈CLS〉 token frequently encountered in the domain of computer vision and natural language processing for classification tasks.^5,40,41^

#### Relevance aggregation

To attribute relevance to the input tokens, we adapted the transformer-specific explainability technique proposed by Chefer *et al.*^33,34^ for computer vision classification tasks to chemical language models using molecular strings and self-attention for regression tasks. The query and key matrices (**Q** and **K**, respectively) of the transformer's attention layers were used to calculate attention scores (**A**) according to eqn (1) (*d*_*k*_ is the dimension of **K**).^4^1
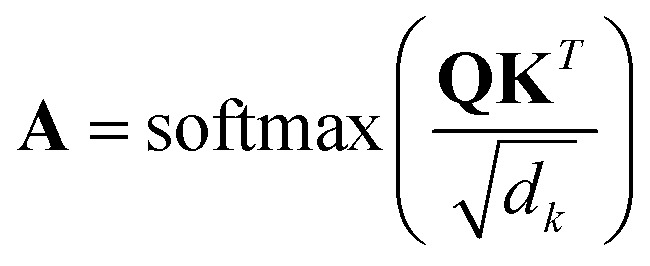


During the model's forward call, attention scores (**A**^*l*^) from all six transformer layers (*l* ∈ *L*) are saved. During the backpropagation step, the gradients 
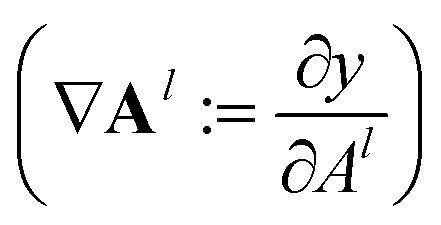
 are evaluated and saved. Each attention head captures different aspects of the task, and the importance of each attention head (*h* ∈ *H*) towards the prediction can be quantified from its gradient ∇**A**^*l*^_*h*_. For each layer, the attention scores of each attention head are then multiplied by their gradients and the resulting values are averaged element-wise over all eight attention heads. Only the positive importance (·)^+^ is considered to reflect nonlinearities following deep Taylor decomposition theory.^47^ These calculations are summarised in eqn (2) (⊙ denotes the element-wise product).

2 **Ā**^*l*^ = **E**_*h*_((**A**^*l*^_*h*_⊙∇**A**^*l*^_*h*_)^+^)

To attribute relevance, a relevancy matrix is initialised as the identity matrix (**R**^0^ = *I*^*T*×*T*^, where *T* is the number of tokens in a SMILES string). The aggregated relevance matrix at layer *l***R**^*l*^ is obtained by matrix-multiplication of **Ā**^*l*^ with the previous layers' aggregated relevance matrix **R**^*l*−1^, which contextualizes the attention mechanism. The product of this operation is summed element-wise with **R**^*l*−1^ (reflecting the model's skip connections). This process is applied throughout all layers (*l* ∈ {1, 2, …, 6}) with an update rule according to eqn (3).

3 **R**^*l*^ = **R**^*l*−1^ + **Ā**^*l*^·**R**^*l*−1^

The attributed relevance of each input token is obtained from the row of the final relevance matrix **R**^*L*^ corresponding to the readout token 〈R〉.

#### Extracting attention scores and gradients

The attribution technique is computationally efficient, as only a single forward and backward pass of the model is necessary to obtain the attention scores and gradients, while the propagation and attribution of relevance are negligible in terms of computational cost and memory. To extract the attention scores and gradients, the “transformers.py” file needs to be slightly modified to save the attention scores and attach a “hook” to evaluate the gradients and extract them to calculate attributions. Since these hooks do not need to be present during model training or validation, we extended the “predict” function and enabled tracking gradients for the model parameters as well as for the input token matrix.

#### SHAP

To obtain attributions for the average-pooling MegaMolBART variants, we applied the established, model-agnostic SHAP technique (SHapley Additive exPlanations), which is based on Shapley values from game theory.^23^ This technique calculates input feature importance by repeatedly masking parts of the input and evaluating the resulting change in prediction, and thus does not need to account for the internal architecture of the model. This is done for many input feature combinations, but requires random sampling of coalitions since evaluating all possible combinations is computationally intractable. SHAP is a model-agnostic technique applicable to all models after training, but uses many model calls to obtain an explanation and is significantly slower than the 〈R〉 token approach. SHAP requires the implementation of a custom masker to enable masking of input tokens, since the tokenizer of MegaMolBART cannot process modified, masked input strings without modification.

#### ECFP

ECFP allows for the identification of substructures present in the molecule, but does not provide per-atom attributions for comparison with other techniques. We overcame this by aggregating the regression weight coefficients of the linear regression head of each bit to all constituent atoms of the substructure. For multi-atom substructures, we divided the regression weight evenly among all constituent atoms and aggregate the contribution for each atom individually to ensure that the overall relevance to the prediction remains constant. We observed that almost all regression coefficients are negative for ECFP, which leads to attributions that are almost entirely negative. We were able to overcome this issue by training ECFP-lin on a scaled regression target, specifically using scikit-learn's “RobustScaler” to scale the log(*S*) target by removing the median and scaling variance using the 10th and 90th percentiles for robustness to outliers.

## 3 Results and discussion

### 3.1 Aqueous solubility prediction

Table 1 shows the test set performance of various MegaMolBART-derived models compared to three other recent models, which predict aqueous solubility at 298 K (log(*S*_aq,298 K_)), as reported by Vermeire *et al.*^18,19,48,49^ Fig. S1 and Table S1‡ show the cross-validation results for all model variants and split strategies.

**Table 1:** Comparison of models trained on the AqueousSolu dataset for a random 10% test set (“random”, 1181 molecules), the low-uncertainty test set^18^ (“accurate”, 578 molecules) and a ∼10% scaffold split (“scaffold”, 925 molecules). The test set mean absolute error (MAE) and root mean squared error (RMSE) from the best-performing model measured as the MAE during cross-validation are reported. Abbreviations: MMB: MegaMolBART, ft: fine-tuned, 〈R〉: readout token, avg: average-pooling, lin: linear regression head, hier: hierarchical regression head, ECFP: 512-bit ECFP fingerprints, ECFP-2K: 2048-bit ECFP fingerprints, SVR: support vector regressor, and RF: random forest regressor

Model variant	Random	Random	Accurate	Accurate	Scaffold	Scaffold
	MAE	RMSE	MAE	RMSE	MAE	RMSE
MMB-ft, 〈R〉, lin	0.622	0.877	0.504	0.655	1.001	1.322
MMB-ft, 〈R〉, hier	0.593	0.858	0.474	0.635	0.949	1.270
MMB, 〈R〉, lin	1.293	1.677	1.270	1.646	1.514	1.897
MMB, 〈R〉, hier	1.052	1.394	0.939	1.230	1.311	1.673
MMB-ft, avg, lin	0.595	0.858	0.439	0.588	0.931	1.235
MMB-ft, avg, hier	0.607	0.873	0.445	0.617	0.938	1.262
MMB, avg, lin	1.048	1.391	0.843	1.124	1.281	1.624
MMB, avg, hier	0.808	1.110	0.667	0.865	1.132	1.459
ECFP, lin	1.179	1.592	1.413	1.821	1.912	2.463
ECFP-2K, lin	1.175	1.601	1.052	1.359	1.616	2.075
ECFP, hier	1.147	1.552	1.019	1.313	1.608	2.047
ECFP-2K, hier	0.917	1.272	0.731	0.964	1.384	1.752
ECFP, lin, scaled	1.650	2.120	1.198	1.498	1.736	2.196
ECFP-2K, lin, scaled	1.395	1.793	0.959	1.239	1.641	2.086
ECFP, SVR	0.811	1.193	0.640	0.930	1.350	1.707
ECFP-2K, SVR	0.751	1.111	0.567	0.817	1.259	1.616
ECFP, RF	0.799	1.177	0.675	0.963	1.407	1.803
ECFP-2K, RF	0.757	1.138	0.616	0.883	1.386	1.786
SolProp	**0.49**	**0.75**	**0.34**	**0.49**	—	—
ALOGpS	—	—	0.55	0.79	—	—
SolTranNet	—	—	0.58	0.76	—	—

Three train-test split methods (random, accurate^18^ and scaffold^50^) were investigated for training and evaluating models. For all model variants that achieve competitive accuracy (*e.g.*, MAE < 0.55), we find that models perform best on the “accurate” split due to lower experimental uncertainty, following the trend reported by Vermeire *et al.*^18^ The scaffold split strategy consistently gives the highest test set errors for all model variants.

In general, the predictions of MegaMolBART are outperformed by those of the previously reported graph neural network for solubility by Vermeire *et al.*,^18,48^ which employs an ensemble of directed message passing neural networks.^50^ However, for the accurate train-test split, the fine-tuned MegaMolBART models perform better than both SolTranNet^19^ (based on a transformer model^20^) and ALOGpS (based on an ensemble of shallow neural networks^49^). We consider the performance of the fine-tuned MegaMolBART models to be sufficiently competitive to provide good predictions of solubility.

There are clear differences between the linear and hierarchical regression head architectures used for each model. Models that use a hierarchical regression head achieve lower MAE/RMSE values than those using a linear regression head. This difference is expected due to the higher model complexity of the hierarchical regression head. All linear regression head models perform poorly without fine-tuning, and we thus find it necessary to use a hierarchical regression head to achieve competitive accuracy for all frozen MegaMolBART variants, as well as for ECFP based molecular representations. Only the pretrained MegaMolBART model (which uses a “frozen” encoder) with average-pooling and a hierarchical regression head (mmb-avg-hier) achieves good results without fine-tuning. We observe significantly worse performance for the average-pooling variants of MegaMolBART with a linear regression head when we train without a LayerNormalization layer applied after the encoder's output before the linear regression head (see Table S2‡). The SVR and RF regression methods with ECFP both perform better than the linear and hierarchical regression heads. However, they do not perform as well as the fine-tuned MegaMolBART variants.

We find that fine-tuning is beneficial in terms of predictive accuracy in all instances, highlighting the benefit of adjusting the learned representations towards the task at hand instead of using the pretrained CLM “out-of-the-box”. Fine-tuning MegaMolBART models leads to the lowest prediction errors, with both the linear and hierarchical regression heads achieving very similar scores. Fine-tuning the MegaMolBART encoder is necessary when using the 〈R〉 token, since the model is not pretrained to leverage the 〈R〉 token as a readout token and thus performs poorly when the encoder remains frozen. All fine-tuned MegaMolBART variants (both the 〈R〉 token and average-pooling approach) achieve similar errors. Thus, the 〈R〉 token enables the attribution of relevance without a significant loss in predictive accuracy. The simpler, linear model variant also performs comparably to the hierarchical variant. Therefore, we chose to investigate the simpler model based on the linear regression head for the transformer-specific explainability technique based on the 〈R〉 token.

### 3.2 Characteristics of fine-tuned MegaMolBART representations

**Fig. 1:**
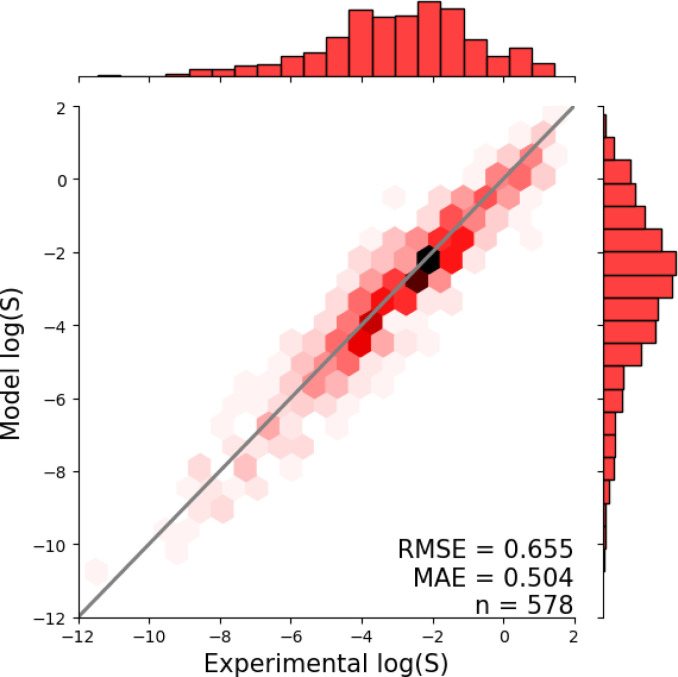
Parity plots showing the predictive accuracy of the MegaMolBART model (mmb-ft-lin) fine-tuned on the AqueousSolu dataset and evaluated on the “accurate” test set. Deviations from the diagonal line represent the model's prediction errors. The predictions are grouped into hexagonal bins to highlight the density of predictions, and the raw densities are shown as histograms on the sides of both plots.

**Fig. 2:**
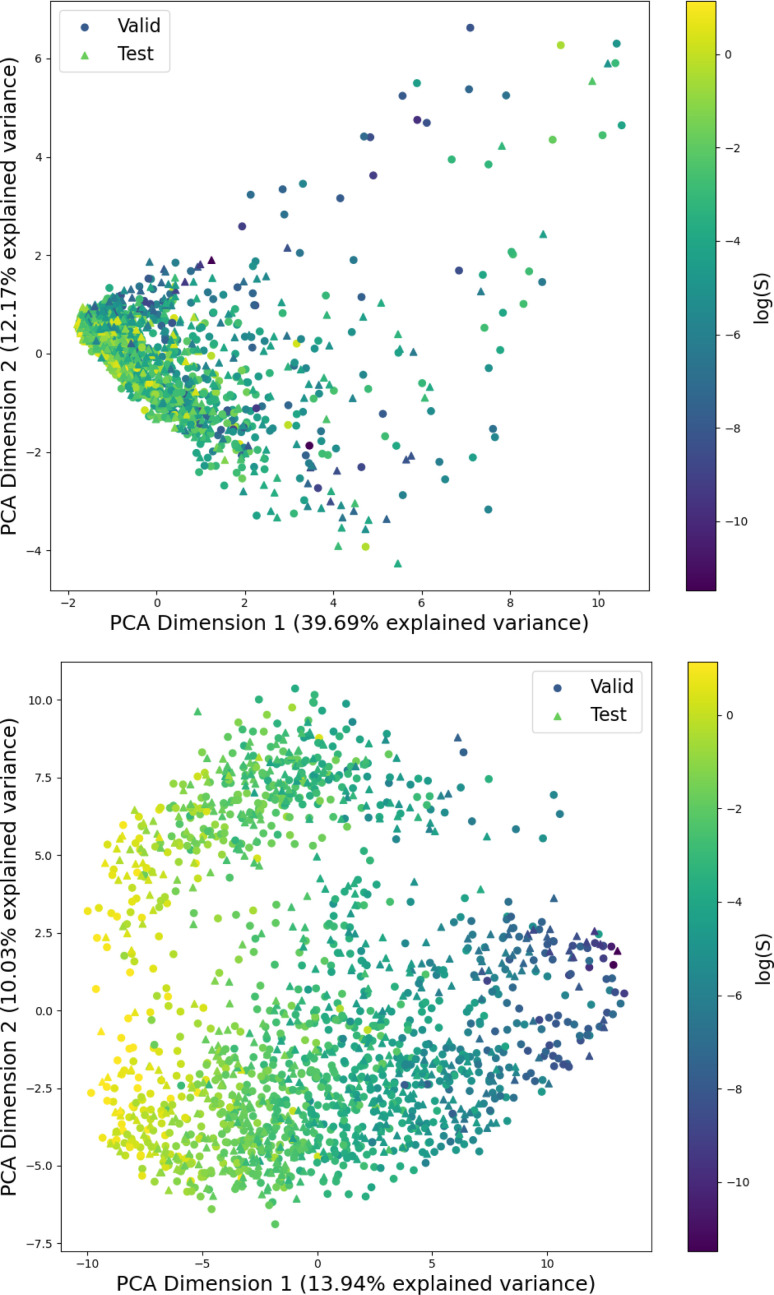
Visualization of the first two principal components obtained from the PCA of the latent space representations. The projection is parametrized on the same validation set for both figures and shows both the validation and test sets of the “accurate” split. Top: The latent representation of the “frozen” average-pooling MegaMolBART model (mmb-avg-hier), which is independent of the regression head when the encoder remains frozen. Bottom: The MegaMolBART model with a linear regression head and the 〈R〉 token (mmb-ft-lin) is fine-tuned to linearly map the latent features to solubility.

Fig. 1 shows the distribution of prediction errors of the fine-tuned MegaMolBART 〈R〉 variant with a linear regression head (mmb-ft-lin) for the “accurate” test set (see Fig. S2‡ for the random test set). The model accurately predicts solubility without extreme outliers, but underestimates log(*S*) for compounds with low solubility. The linear regression head enables us to think of the encoder as a feature extractor, which is mapped linearly to solubility without any further nonlinear projection layers. To illustrate, we compare latent representations produced by the encoder layers of mmb-avg-lin and mmb-ft-lin by projecting the latent representations into two dimensions using their first two principal components fitted on the validation set (Fig. 2). Though the two encoders use a different strategy (average-pooling *vs.* the 〈R〉 token) to obtain an aggregate representation suitable for the regression head, we consider this a more valid comparison than between the frozen and fine-tuned 〈R〉 model variants, since fine-tuning is necessary to train the model to leverage the 〈R〉 token. The projections of latent representations produced by the pretrained model using an average-pooling strategy (Fig. 2, top) indicate that the latent space of the model has no discernible patterns for solubility. Strikingly, the latent representations of the fine-tuned variants (Fig. 2, bottom and Fig. S3‡) show a continuous decrease in log(*S*) along the first principal component, indicating a chemical space that is well-organised for mapping to solubility.

### 3.3 Explaining the predictions of MegaMolBART

**Fig. 3:**
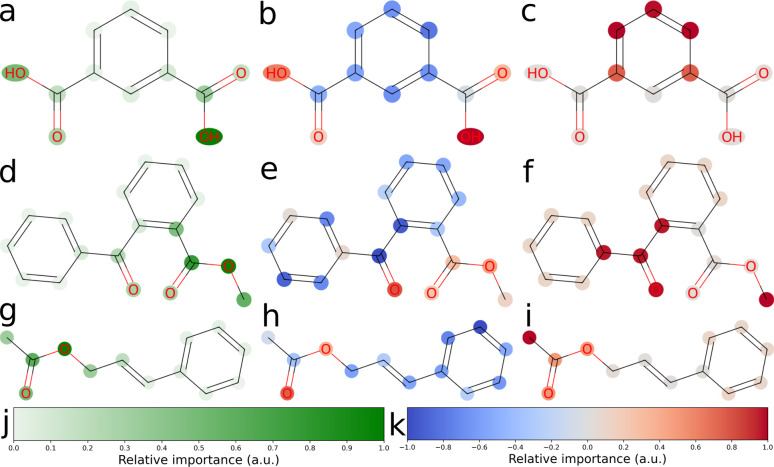
2D structure plot corresponding to attributed relevance for three molecules in the “accurate” test set. The relevance of each atom token to the model's aqueous solubility prediction is visualized. (a), (d) and (g) Attributions obtained from the fine-tuned MegaMolBART model using the 〈R〉 token and a linear regression head (mmb-ft-lin). (b), (e) and (h) Attributions obtained from SHAP explaining the fine-tuned average-pooling MegaMolBART model and with a linear regression head (mmb-ft-avg-lin). (c), (f) and (i) Attributions obtained from ECFP with a linear regression head (ecfp-lin-scaled) by attributing regression weights to all atoms of the bits' substructure uniformly, trained with a scaled log(*S*) target. (j) The green color bar shows relative importance in arbitrary units, which is only positive for MMB explanations using the 〈R〉 token. (k) The diverging color bar used by SHAP and ECFP uses cool (blue) and warm (red) for negative and positive attributed relevance, respectively.

We explored three approaches to developing explainable models for solubility predictions. SHAP and a gradient-based attribution method were used to produce explanations of MegaMolBART's predictions of aqueous solubility. As an algorithmic, “explainable” comparison, we also derived explanations from ECFPs using the linear regression head weights. Bit vectors of sizes 512 and 2048 were investigated for their predictive performance. Here, we have focused on the explainability of the 512 bit vector size, which reflects the dimensionality of latent representations produced by MegaMolBART. Solubility values were centered and normalised so that the regression head weights would directly reflect contributions towards higher (positive weights) or lower (negative weights) than median solubility. Representative atomic attributions derived from all three explainability methods are shown in Fig. 3 and S6–S9.‡ Attributions were selected for inspection by sampling from different regions of the dimension-reduced latent space outlined in Fig. 2 (bottom), using *k*-means clustering with *k* = 4 (Fig. S4 and S6–S9‡).

We selected the twelve compounds closest to each cluster centroid to visualise the explanations obtained from MegaMolBART using the 〈R〉 token (Fig. S4 and S10–S13‡). The attributions derived from mmb-ft-lin appear to provide consistent attributions to atoms among similar compounds. For instance, atomic features selected by mmb-ft-lin include chlorine atoms bound to aromatic rings, single oxygen atoms bound to carbons (both alcohols and carbonyls), carboxylic acid/ester groups and single nitrogen atoms (Fig. S10–S13‡). Inspecting the attributions from atomic tokens, we find that they are relatively sparse in comparison to those provided by SHAP and ECFP (Fig. S6–S9‡). The consistency of certain features suggests that MegaMolBART is selecting specific, general components of SMILES strings to create molecular representations.

**Fig. 4:**
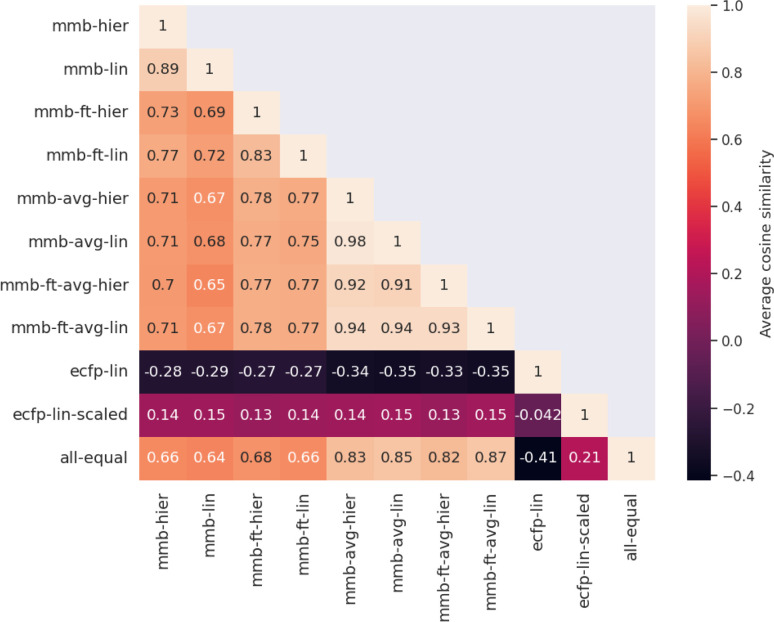
Quantitative comparison of attributed atom relevance for the “accurate” test set. For each model pair, the cosine similarity of attributed relevance is calculated as the average over all molecules in the test set. The first four MegaMolBART models use the 〈R〉 token and attribution method adapted in this work, while the middle 4 MegaMolBART models use the average-pooling approach in combination with SHAP to obtain attributions. For both models, fine-tuning (-ft-) or frozen strategies and regression head variants (lin or hier) are compared. ECFP attributions are extracted by attributing regression weights to all constituent atoms and aggregating per-atom contributions. We additionally compare attributions from ECFP trained on a scaled regression target (ecfp-lin-scaled), since the attributions of ecfp-lin are mostly negative because most regression coefficients have negative signs. The last entry shows uniform relevance, a baseline where every atom has the same normalized attributed importance towards the prediction.

The sparsity of the explanations inferred from MegaMolBART is consistent with the attention mechanism providing a strong degree of feature selection for the construction of the encoded, latent representations. Despite this, the features that are being selected do not appear to be directly related to solubility (for example, the aforementioned C–Cl bonds). Rather, the derived attributions appear to be more suggestive of key features relevant to how MegaMolBART determines molecular similarity, and thus are more pertinent to the organisation of the model's latent space. If this is the case, the fine-tuned MegaMolBART encoder and regression head can be conceptualised as a true molecular representation generator/regressor pair. Furthermore, the MegaMolBART model learns to predict solubility accurately without fully learning a relevant physical context for solubility. This behaviour is consistent across different fine-tuning and regression head variants, which all produce quantitatively comparable results and qualitatively similar visualizations (Fig. 4). We hypothesize that such feature selection is due to the major influence of the pretrained MegaMolBART model. Atoms with high attributed relevance generally correspond to parts of relevant functional groups for aqueous solubility (such as OH, =O or NH_2_), while explanations deteriorate for molecules with very high or low solubility. The model is unable to accurately model symmetry and frequently attributes very different relevance to symmetric functional groups, which might be due to the difficulty of reconstructing the structure of the molecule from a string-based representation.

Visualising the attributions provided by SHAP shows clear positive contributions from single electronegative atoms such as oxygen and nitrogen, as well as negative contributions from carbon skeletons (Fig. 3b, e, h and S6–S9‡). Thus, SHAP is able to accurately probe the chemical language model to obtain chemically sensible atomic attributions. However, SHAP does not give any insight into how the CLM represents molecular strings or how it obtains its prediction. Furthermore, the assumptions of this technique, such as the additivity of features and fair coalitions, are not valid for chemical language models because they operate on molecular string representations. The omission of some tokens frequently leads to invalid molecules, as these tokens directly represent the molecules' physical structure and connectivity between atoms. Determining valid substitution rules is very difficult for molecules, and most substitutions would significantly change the predicted property. Similarly, functional groups might be a good fit to treat as coalitions, but would require modifications to how SHAP values are calculated when sampling is not random, in addition to the need to determine which functional groups should be considered. SHAP can be applied to models for natural language, but its application to tokenized molecular strings is analogous to masking out random letters of a single word and evaluating the importance of each letter.

Neither SHAP nor the gradient-based attribution method provides scope for the inference of contributors to solubility at the functional group level. If motifs of collections of atoms are present, they do not occur often between neighbours.

In contrast to the explanations inferred from MegaMolBART, the ECFP model is more eager to assign importance to larger subgroups of atoms, as is expected from the Morgan fingerprinting algorithm (Fig. 3c, f, g and S6–S9‡). It could thus be expected that they provide better handles for understanding solubility at the functional group level. However, this level of “coarse graining” provided by the 512 bit fingerprint clearly comes at a cost of model performance for predictive accuracy, implying that the “explainable” features generated by the fingerprinting algorithm are poorly suited to predicting solubility. Indeed, this is consistent with the better performance of models using an ECFP bit vector size of 2048 (Table 1). We find that the explanations inferred from the ECFP model are not satisfactory. Often, predominantly carbon-based substructures are attributed, such as aromatic rings and methyl groups. This result likely arises due to so-called bit-collisions occurring in the Morgan fingerprinting algorithm, which result in the attribution of two atomic environments to the same feature. This phenomenon is likely to be particularly severe in our case due to the size of the fingerprint chosen (512 bits). The resulting collisions likely mask the “true” feature contributions, negatively impacting explainability. As such, the compression entailed in creating a 512 dimensional ECFP representation is too severe to retain the explainable properties of the model. However, the explanations gained from a 2048 bit vector size are similarly coarse-grained and not much more informative than those from the 512 bit vector fingerprints (Fig. S14–S17‡). Thus, the unsatisfactory explainability from this method may simply be due to the unsuitability of the underlying fingerprinting algorithm for capturing soluble functional groups. Given the moderate success of the SHAP attribution method, the compressed representations learned by fine-tuned MegaMolBART may be more explainable and certainly better predictors than an ECFP method of similar dimensionality.

### 3.4 Comparisons of attributed relevance between methods

Evaluating which attribution is correct or most accurate is not possible since no reference attribution label exists for solubility or other physical properties. We instead compare the attribution results quantitatively with respect to each other by measuring the cosine similarity of attributed relevance between model pairs.

Fig. 4 shows the average cosine similarity of the attributions obtained from each model pair for the “accurate” test set (see Fig. S18‡ for the random test set). Regions of high cosine similarity can be seen for all attributions obtained from the average-pooling MegaMolBART model and its variants. All attributions are highly similar due to the way SHAP calculates its explanations, which show little influence from the regression head or fine-tuning. In contrast, the similarity in attributed relevance is significantly lower and more varied among the MegaMolBART models based on the 〈R〉 token. The frozen variants show high similarity, but the similarity between those variants as well as compared to SHAP attributions is lower. The attributions obtained from both explainability approaches for MegaMolBART vary among all models that achieve competitive accuracy on the test set, as measured by ≥0.77 cosine similarity (mmb-ft-lin, mmb-ft-hier, mmb-ft-avg-lin, mmb-ft-avg-hier, and mmb-avg-hier).

### 3.5 Positive and negative contributions

Given the chemists' intuition of hydrophilic and hydrophobic groups, we extend our attribution technique to enable the attribution of individual features that contribute positively (more soluble) or negatively (less soluble) to the CLM's solubility predictions. We achieve this by masking all but one latent feature using the same fine-tuned model in the forward and backward passes, effectively constraining the model to predict using only one of the latent features at a time. We highlight the difference between masking the latent features compared and masking the input tokens, as done in pretraining strategies or SHAP. The masked regression prediction thus corresponds to the isolated contribution of that feature, and our modified attribution technique highlights which input tokens contributed to this particular latent feature.

We fail to discover interpretable features using this approach and focus on separately aggregating all features that have a positive or negative contribution to the overall prediction. In the context of solubility, this could correspond to a separation into hydrophilic and hydrophobic parts of the molecules. We apply the same approach, masking out all latent features that have an overall positive contribution to aggregate only negative attributions and repeat this procedure for the opposite sign. We emphasize the necessity of considering the sign of the product between the activation and the regression weight of the linear regression head. This divides features into four types of contributions, based on the combinations of the sign of the activation and the sign of the regression coefficient (see Fig. S19‡). Features with the same sign {(+, +), (−, −)} have an overall positive contribution to the prediction, while features with opposing activation and regression signs {(+, −), (−, +)} have a negative contribution. Compared to the attribution of individual features that require one model call per feature, we can obtain attributions for all positive or negative contributions in one model call for each sign.

We discover that only features with a positive contribution can be attributed to the input with our adapted attribution technique leveraging the 〈R〉 token, while features with negative contributions show zero attributed relevance in isolation. We trace the source of this behaviour to the gradients of the attention heads (∇**A**^*l*^_*h*_), which are all-zero for features that have a negative contribution to the prediction (see Fig. S20‡). This prevents the propagation of the gradient throughout the layers and thus yields all-zero contributions towards the layer's aggregated relevance matrix **Ā**^*l*^. We refer to Section S10 and Fig. S19 and S20‡ for a detailed explanation and discussion as well as visualizations of the isolated contributions of the masked attention heads. Features with an overall negative contribution towards the prediction thus have zero contribution towards the explanation, but those features constitute a significant part of the prediction. We hypothesize that this limitation is one of the reasons why the explanations of this attribution method do not correspond to the hydrophobic and hydrophilic atoms or functional groups of the molecule.

Many attribution methods find theoretical justification in the work of Montavon *et al.*,^47^ which proposes deep Taylor decomposition. The authors define heatmaps as “consistent” if they fulfil the conservation of relevance and yield only positive values without negative relevance. The conservation of relevance requires the overall attributed relevance to be approximately equal to the prediction, and thus for overall relevance to remain constant between layers. Positive activations are relevant since deep learning models use nonlinearities such as ReLU, which shifts negative inputs to 0 and only keeps positive activations. The authors define a training-free relevance model using the *z*^+^ rule for architectures using ReLU nonlinearities, which have positive activations. This enables the propagation of relevances in higher layers in proportion to the model's activations and decomposes the architecture layer by layer to the input.^47^ While this theoretical justification is not explicitly stated, the assumption of positive relevance is implicit in many attribution techniques.^51^ Sixt *et al.*^52^ analyzed many established and recent attribution methods, highlighted this aspect as a significant limitation and showed that methods that only propagate positive relevance collapse toward a linear subspace with each additional layer.

## 4 Conclusion

In this work, we explored the use of chemical language models for explainable property prediction. Our model comparison shows that pretrained MegaMolBART models achieve competitive accuracy in predicting aqueous solubility, and visualizations of the fine-tuned model show a linearly separable latent space for solubility. Fine-tuning the encoder in addition to the regression head is beneficial for all models and necessary in our modified MegaMolBART variant leveraging a readout (〈R〉) token.

Though ECFP is capable in theory of providing molecular features that are directly attributable to molecular structures, we find that regressing ECFP based representations onto log(*S*) cannot compete with state-of-the-art models. Furthermore, the algorithm underlying the construction of ECFPs may not capture features relevant to solubility, thus impacting its performance as a method for making chemical inferences from models derived from it. SHAP is able to accurately probe the chemical language model to obtain sensible chemical attributions, such as placing positive relevance on single electronegative atoms and negative contributions for the carbon skeleton. However, SHAP does not enable any inference of contributors to solubility at the level of functional groups.

The transformer-specific XAI technique adapted to explain the CLM in regression tasks produces sparse attributions with the most relevance attributed to a small subset of tokens. These attributions appear to be more a signature of how the fine-tuned models produce molecular representations that are more linearly separable and tuned for regression onto solubility. As a result, the adapted attribution technique leveraging the 〈R〉 token is better for selecting specific features that distinguish structures relative to one another, as opposed to selecting sets of features that reflect a subtle balance between substructures that contribute positively to solubility and those that contribute negatively to solubility. We hypothesise that this behaviour may be due to the inability of the attribution method to propagate negative gradient information from the regression head to the attention heads. The visualizations of the model's latent space lead us to conclude that the model uses SMILES strings to map molecules into a structural latent space and predicts solubility based on position, rather than regressing based on learned molecular features and functional groups.

We hypothesise that the attributions obtained by explaining MegaMolBART with the 〈R〉 token approach could be used as a handle for the development of focused, informative chemical data sets. By understanding which structures the model focuses on for mapping solubility, structural modifications can be applied, which contravene the model's predictions to create new training examples. For instance, attributed features from an insoluble compound could be introduced into a soluble compound so as to make it insoluble. Measuring the solubility of this compound and adding it to the training data would thus contribute to creating a more diverse chemical space, as well as refining the attribution explanations provided by the model.

## Data availability

(1) The MegaMolBART model (tag v0.2.0) used in this work was cloned from GitHub: https://github.com/NVIDIA/MegaMolBART, and is available on the official NVIDIA website: https://catalog.ngc.nvidia.com/orgs/nvidia/teams/clara/containers/megamolbart_v0.2, tag 0.2.0. (2) All training, inference and explainability code developed in this work is available on GitHub: https://github.com/BigChemistry-RobotLab/explaining_a_chemical_language_model. (3) All solubility data used in this manuscript are available on Zenodo: https://zenodo.org/records/5970538 (cited as *J. Am. Chem. Soc.*, 2022, **144**(24), 10785–10797).

## Author contributions

S. H. developed the methodology and code for model training and attributions. S. H. and W. E. R. wrote and edited the manuscript. W. E. R. and W. T. S. H. supervised the project. S. H., W. E. R., W. T. S. H. and T. K. conceptualized the project. W. T. S. H. acquired funding. All authors reviewed and discussed the manuscript.

## Conflicts of interest

There are no conflicts to declare.

## Supplementary Material

DD-003-D4DD00084F-s001
