# Supplementary material for: What can attribution methods show us about chemical language models?
Source: Digit Discov. 2024 Jul 18;3(9):1738–48. doi: 10.1039/d4dd00084f (PMC11389796; doi:10.1039/d4dd00084f)
Supplement: DD-003-D4DD00084F-s001 [file DD-003-D4DD00084F-s001.pdf]

Electronic Supplementary Material (ESI) for Digital Discovery.
This journal is © The Royal Society of Chemistry 2024

Journal Name

ARTICLE TYPE

Cite this: DOI: 00.0000/xxxxxxxxxx

Electronic Supplementary Information for

What can Attribution Methods show us about Chemical Language Models?

Stefan Hödl,[a] Tal Kachman,[b] Yoram Bachrach,[c] Wilhelm T.S. Huck[a] and William E. Robinson[a*]

[a] *Physical Organic Chemistry, Radboud University, Heyendaalseweg 135, 6525AJ Nijmegen, The Netherlands*
[b] *Artificial Intelligence, Donders Institute, Radboud University, Thomas Van Aquinostraat 4, 6525GD Nijmegen, The Netherlands*
[c] *Google Deepmind*
[*] *Corresponding author: william.robinson@ru.nl*

1 Visualizations of the “random”, “accurate” and “scaffold” validation/test set error

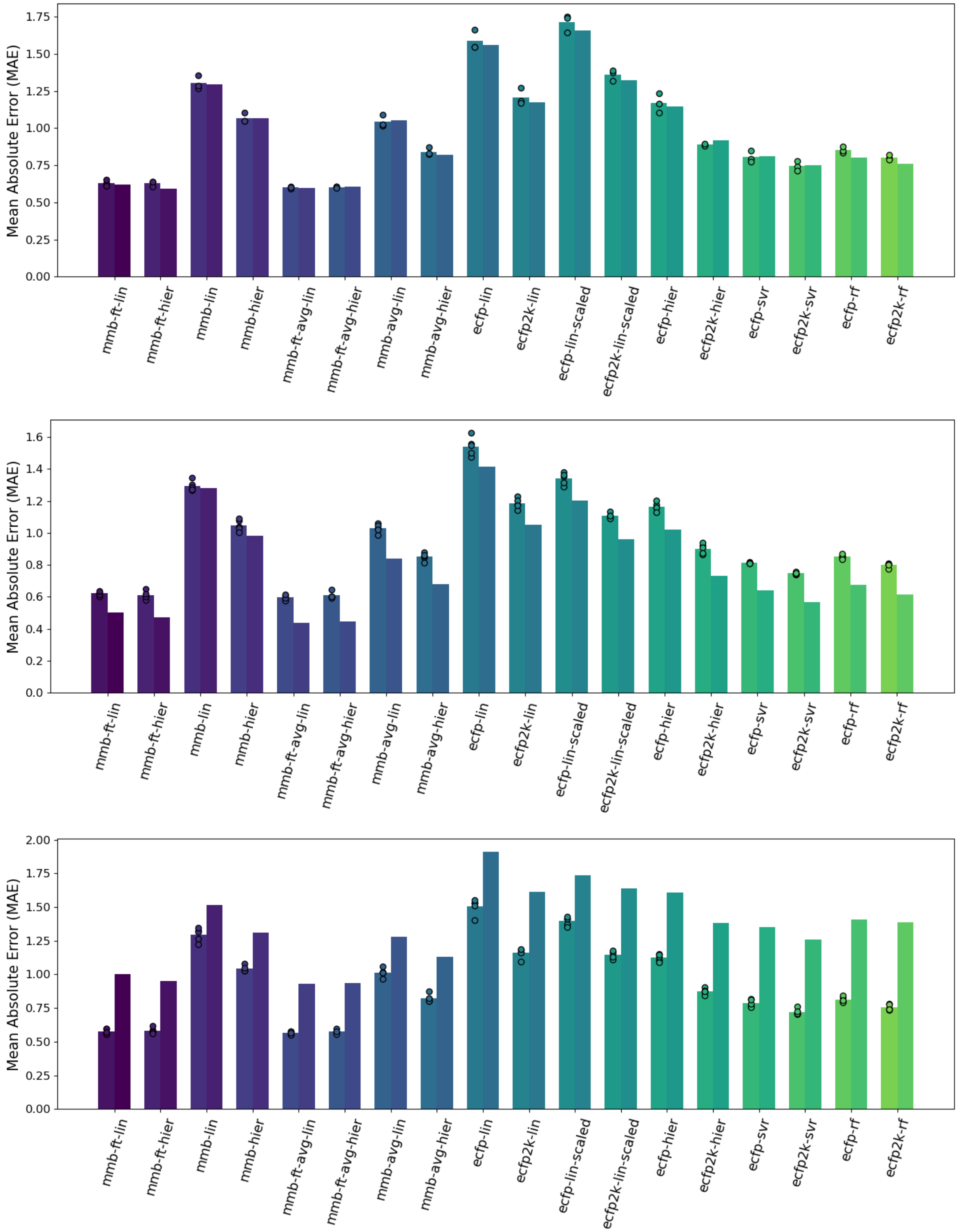

Fig. S 1 Bar chart comparing the predictive errors of different model variants for the “random” test set (top), “accurate” test set (middle) and “scaffold” test set (bottom). The mean absolute error (MAE) of the accurate test set is reported, showing the average cross validation (left bars) and test set error (right bars) from five random splits. All cross-validation errors for each model are plotted as individual dots on the left bar (validation set). We compare the MMB variant with the readout token (<R>) approach (first four models) with average-pooling (avg) (middle four models). We differentiate between keeping the MMB encoder frozen or finetuning it (-ft), and compare the hierarchical regression head (hier) with a simplified, linear regression head (lin) for all model variants. The last two models are ECFP with a hierarchical and a linear regression head.

2 Cross-validation errors for validation set of "random", "accurate" and "scaffold" split strategy

Table S 1 Comparison of models trained on the AqueousSolu dataset. All validation sets are split randomly after the test set has been excluded. The average (mean) and standard deviation (std) of the validation set mean absolute error (MAE) from three (random) or five (accurate, scaffold) cross-validation splits are reported. Abbreviations: MMB: MegaMolBART, lin: linear regression head, hier: hierarchical regression head, ft: finetuned, avg: average-pooling, ecfp: 512-bit ECFP fingerprints, ecfp2k: 2048-bit ECFP fingerprints, svr: support vector regressor, rf: random forest regressor.

Model Variant	Random CV mean MAE	Random CV std MAE	Accurate CV mean MAE	Accurate CV std MAE	Scaffold CV mean MAE	Scaffold CV std MAE
mmb-ft-lin	0.631	0.018	0.621	0.011	0.577	0.018
mmb-ft-hier	0.628	0.014	0.612	0.024	0.583	0.018
mmb-lin	1.302	0.037	1.293	0.028	1.294	0.046
mmb-hier	1.067	0.026	1.048	0.030	1.043	0.021
mmb-ft-avg-lin	0.600	0.006	0.596	0.014	0.566	0.011
mmb-ft-avg-hier	0.602	0.003	0.609	0.018	0.575	0.013
mmb-avg-lin	1.043	0.032	1.028	0.026	1.014	0.029
mmb-avg-hier	0.841	0.021	0.851	0.021	0.825	0.025
ecfp-lin	1.585	0.055	1.541	0.053	1.505	0.052
ecfp2k-lin	1.207	0.046	1.185	0.030	1.160	0.033
ecfp-lin-scaled	1.711	0.050	1.339	0.033	1.432	0.030
ecfp2k-lin-scaled	1.359	0.030	1.109	0.014	1.177	0.025
ecfp-hier	1.168	0.053	1.164	0.024	1.126	0.022
ecfp2k-hier	0.889	0.006	0.901	0.029	0.874	0.020
ecfp-svr	0.804	0.031	0.813	0.003	0.786	0.024
ecfp2k-svr	0.745	0.027	0.747	0.005	0.722	0.021
ecfp-rf	0.853	0.018	0.852	0.011	0.814	0.020
ecfp2k-rf	0.803	0.014	0.799	0.012	0.755	0.019

3 Ablation results for linear regression head without LayerNorm

Table S 2 Comparison of models based on the linear regression head trained with and without a "LayerNorm" normalization layer applied before the linear regression head. All models are trained on the AqueousSolu dataset for the low-uncertainty test set ("Accurate", 578 molecules). The test set mean absolute error (MAE) and root mean squared error (RMSE) from the best-performing model during cross-validation with 5 different random splits are reported.

Model Variant	Accurate MAE	Accurate RMSE
MMB-ft, <R>, lin	0.516	0.669
MMB-ft, <R>, lin, LN	0.504	0.655
MMB, <R>, lin	1.399	1.838
MMB, <R>, lin, LN	1.270	1.646
MMB-ft, avg, lin	1.363	1.820
MMB-ft, avg, lin, LN	0.439	0.588
MMB, avg, lin	1.737	2.245
MMB, avg, lin, LN	0.843	1.124
ECFP, lin	2.122	2.730
ECFP, lin, LN	1.413	1.821
ECFP, lin, scaled	1.296	1.639
ECFP, lin, scaled, LN	1.515	1.907

4 Visualizations for the "random" test set

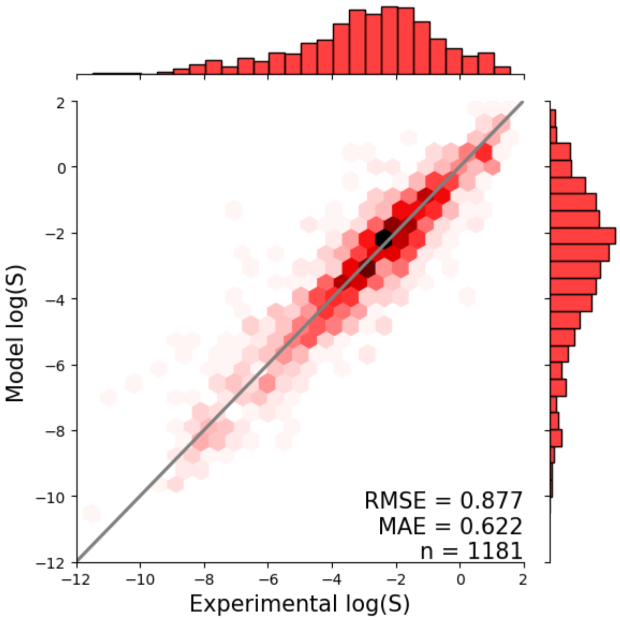

Fig. S 2 Parity plots showing the predictive accuracy of the MegaMolBART model with a linear regression head and the <R> token (mmb-ft-lin) finetuned on the AqueousSolu dataset and evaluated on the "random" test set. The experimental solubility measurements $\log(\mathrm{S})$ (X axes) are plotted against the models' predictions (Y axes), deviations from the diagonal represent the models' errors. The predictions are grouped into hexagonal bins to highlight the density of predictions, the raw densities are shown as histograms on the sides of both plots. The final model is selected based on the lowest validation MAE obtained from cross validation from 3 random validation splits.

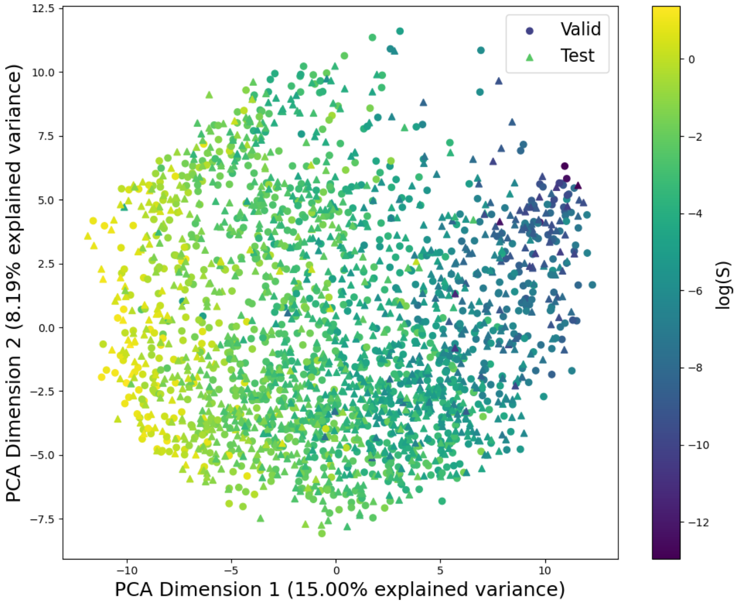

Fig. S 3 PCA visualization of the latent space of finetuned MegaMolBART model with a linear regression head and the <R> token (mmb-ft-lin). The model is finetuned to linearly map the latent features to solubility. A continuous decrease in $\log(\mathrm{S})$ can be seen along the first principal component.

5 K-means cluster visualizations of PCA latent space

To showcase a wider range of molecules for comparison of the similarity of attributions for different regions in the latent space, we plot the neighborhood of representative clusters obtained from KMeans clustering. Specifically, we parametrize the PCA projection on the validation set latent vectors, and use k-means (k=4) to select clusters in the "accurate" test set. We then select the 12 nearest

neighbours in the test set based on distance from the cluster centroid in 2d PCA space.

We visualize the cluster centroids on the PCA latent space visualization below and show the neighborhoods for representative clusters in Figures S10 to S13. In the following figures, we showcase molecules from the same clusters for comparison of the attributions of different explainability techniques (Figures S6 to S9).

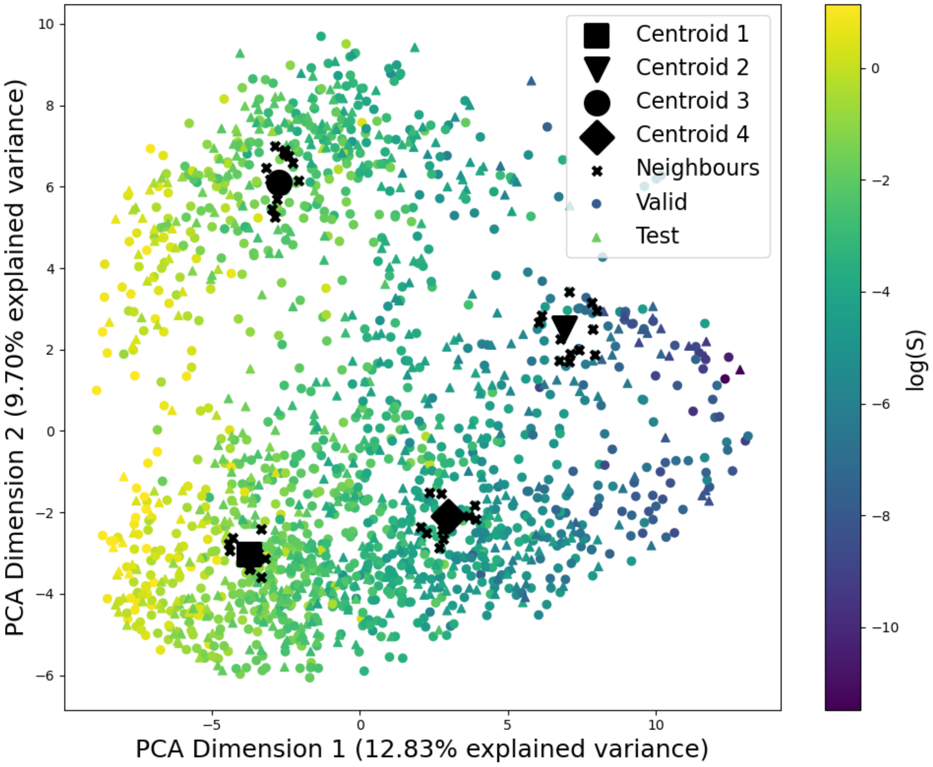

Fig. S 4 PCA visualization of the latent space of finetuned MegaMolBART model with a linear regression head and the <R> token (mmb-ft-lin). We parametrize the PCA projection using the validation set and apply k-means clustering on the test set to obtain cluster centroids. We use k=16 for both PCA and k-means clustering, showing the cluster centroids with black X markers and highlighting those clusters visualized with blue X markers. We then evaluate the neighborhood from these clusters for visual comparison of the attributions among the neighborhood of molecules in latent space.

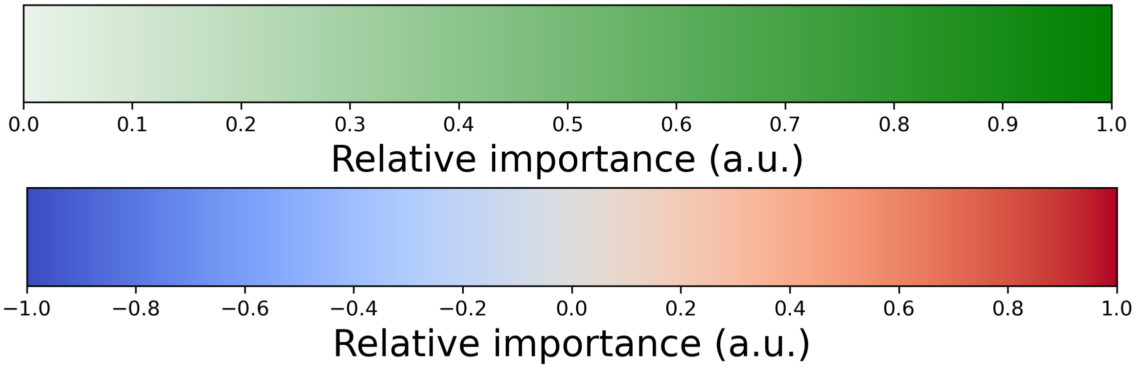

Fig. S 5 Two colorbars, respectively the green colorbar for visualizations based on the <R> token which only have positive relevance, and the diverging colormap (cool/warm) for attributions obtained from SHAP and ECFP.

6 Comparison of attributions from all explainability techniques

To enable comparison between the explanations of all three explainability techniques, we show molecules from the same clusters with attributions obtained from the MMB <R> variant (mmb-ft-lin, left), SHAP (mmb-ft-avg-hier, center) and ECFP (ecfp-lin-scaled, right).

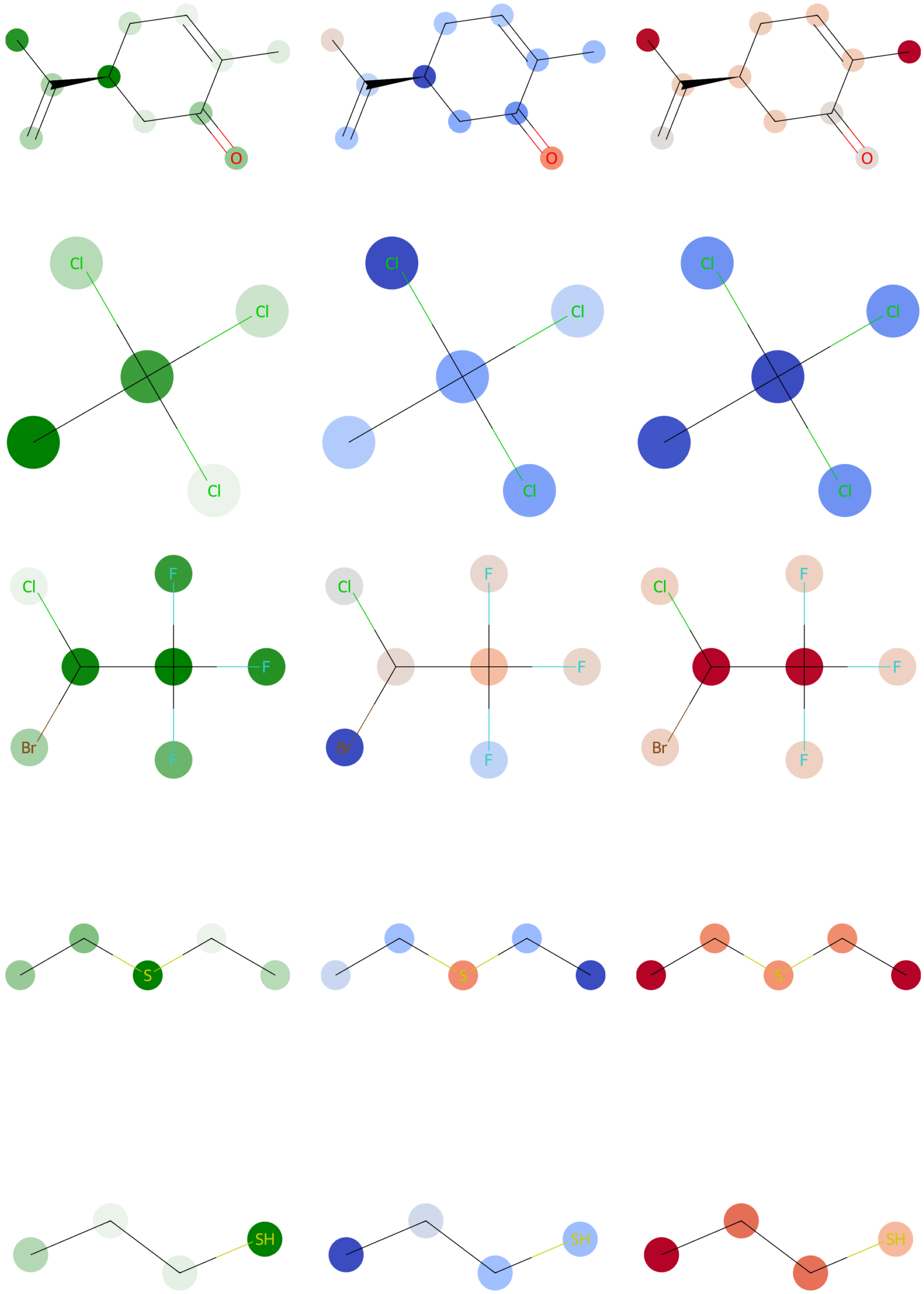

Fig. S 6 Five neighboring molecules from PCA cluster one (Fig. S4) are explained, comparing all three explainability techniques. Left: MMB <R> variant (mmb-ft-lin), Middle: SHAP (mmb-ft-avg-hier), Right: ECFP (ecfp-lin-scaled). The green colorbar is used for the <R> MMB model (Fig. S5, top), a diverging colormap (Fig. S5, bottom) is used to visualize SHAP and ECFP.

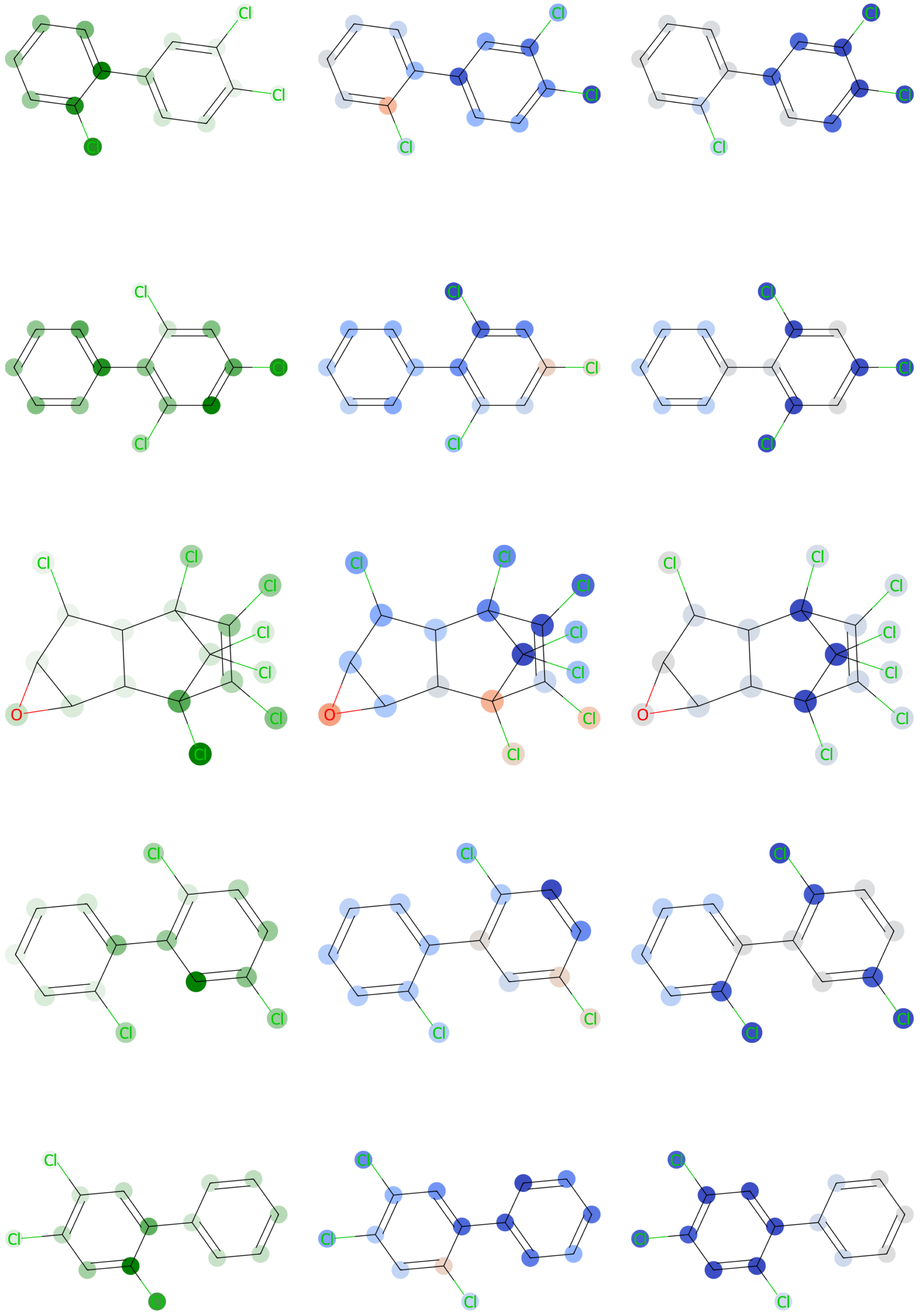

Fig. S 7 Five neighboring molecules from PCA cluster one (Fig. S4) are explained, comparing all three explainability techniques. Left: MMB <R> variant (mmb-ft-lin), Middle: SHAP (mmb-ft-avg-hier), Right: ECFP (ecfp-lin-scaled). The green colorbar is used for the <R> MMB model (Fig. S5, top), a diverging colormap (Fig. S5, bottom) is used to visualize SHAP and ECFP.

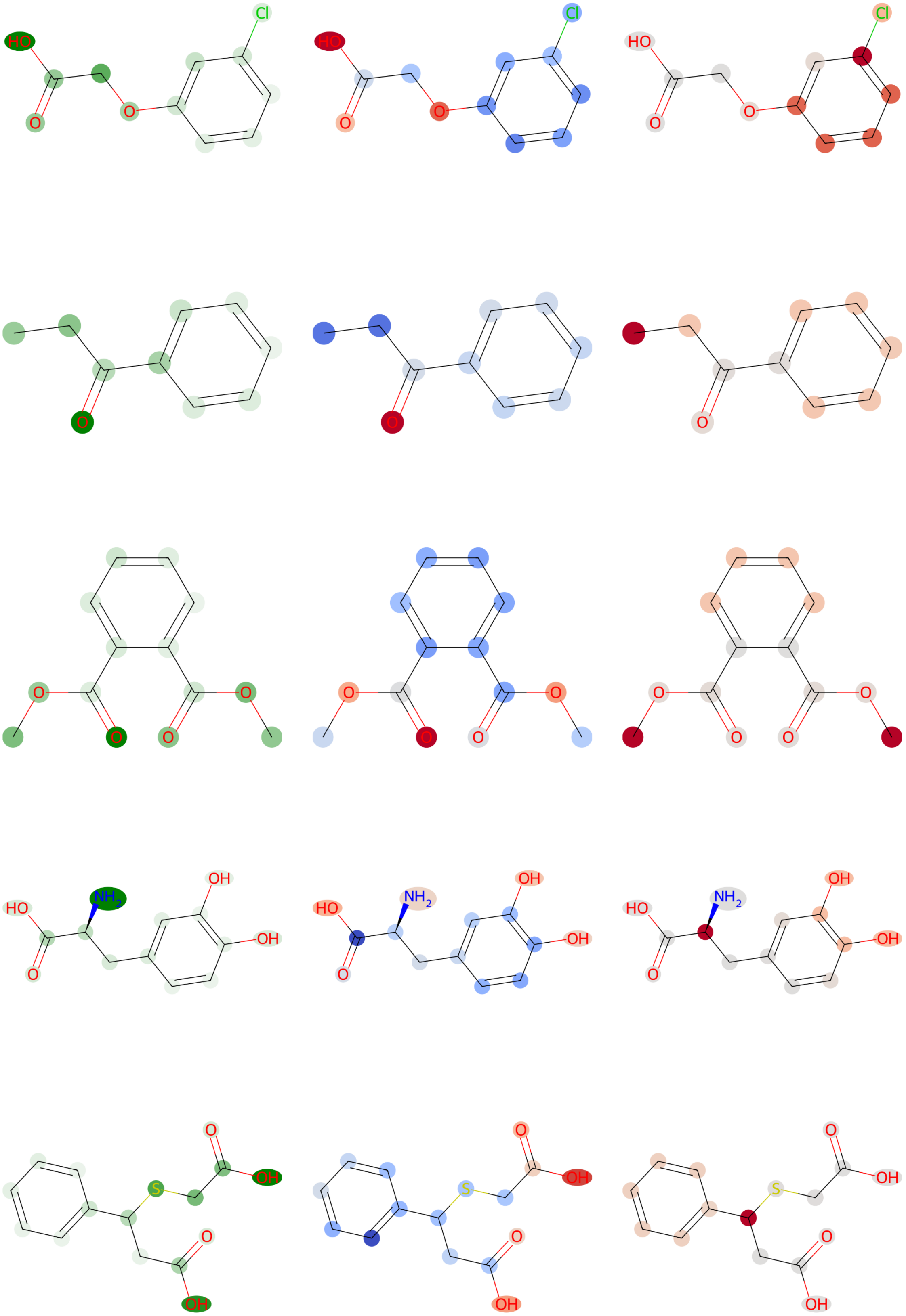

Fig. S 8 Five neighboring molecules from PCA cluster one (Fig. S4) are explained, comparing all three explainability techniques. Left: MMB <R> variant (mmb-ft-lin), Middle: SHAP (mmb-ft-avg-hier), Right: ECFP (ecfp-lin-scaled). The green colorbar is used for the <R> MMB model (Fig. S5, top), a diverging colormap (Fig. S5, bottom) is used to visualize SHAP and ECFP.

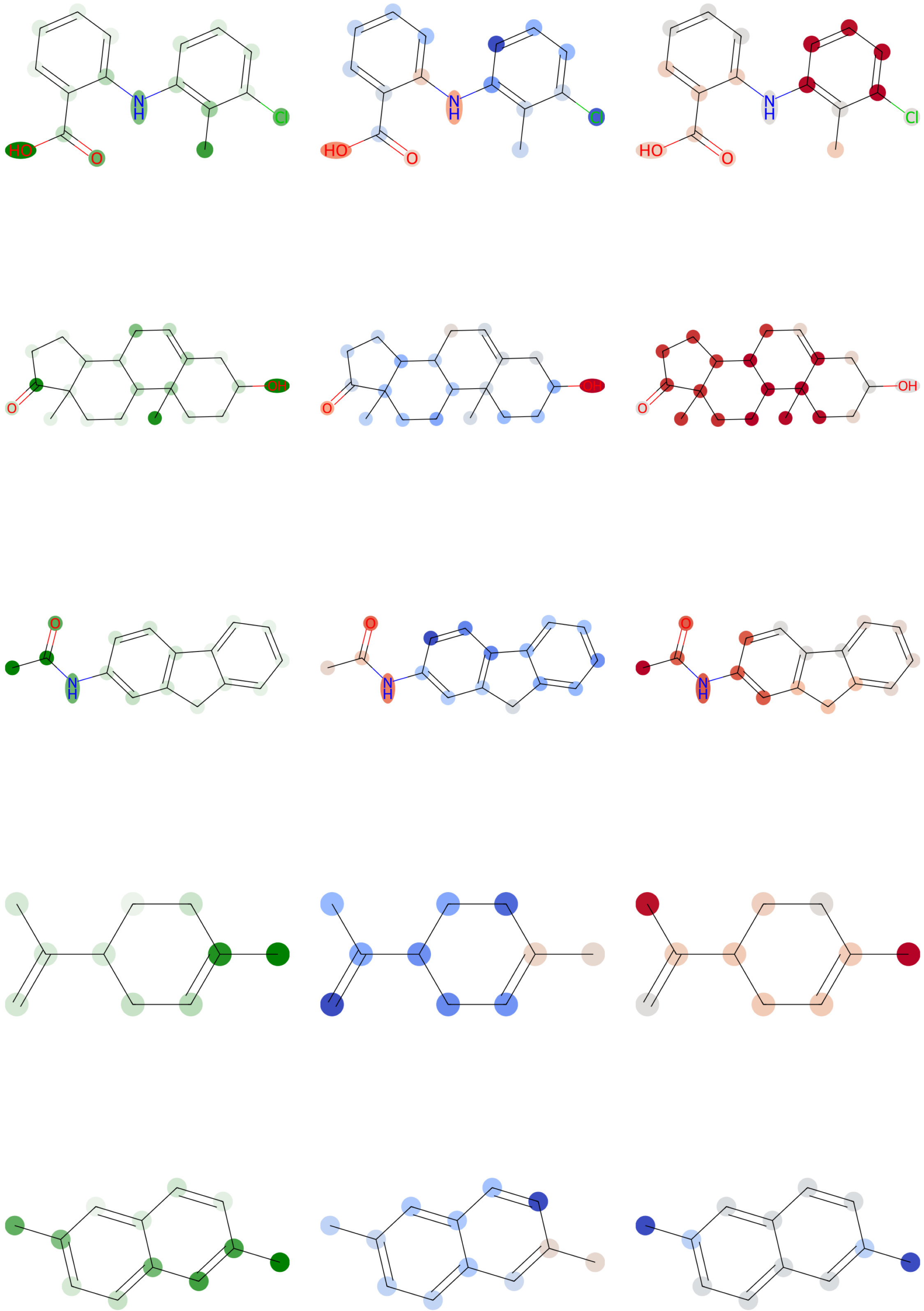

Fig. S 9 Five neighboring molecules from PCA cluster one (Fig. S4) are explained, comparing all three explainability techniques. Left: MMB <R> variant (mmb-ft-lin), Middle: SHAP (mmb-ft-avg-hier), Right: ECFP (ecfp-lin-scaled). The green colorbar is used for the <R> MMB model (Fig. S5, top), a diverging colormap (Fig. S5, bottom) is used to visualize SHAP and ECFP.

7 MegaMolBART explanations from PCA cluster neighborhoods

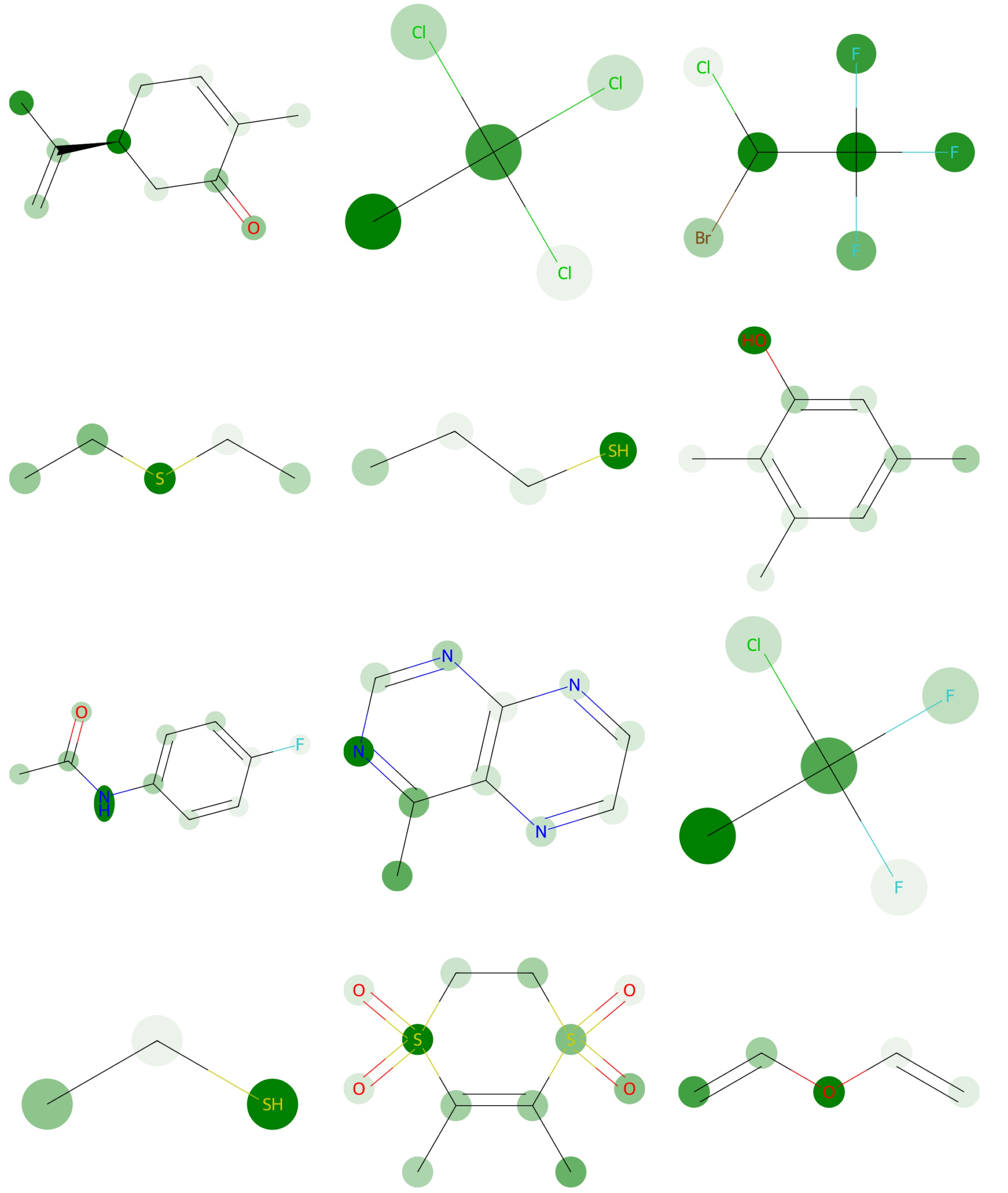

Fig. S 10 Visualizations of the molecular neighborhood of cluster one, obtained through k-means (k=4) clustering on the 2d PCA-reduced embeddings (Fig. S4) and selecting the nearest neighbors based on 2d PCA distance. The corresponding colorbar can be seen in Fig. S5, top.

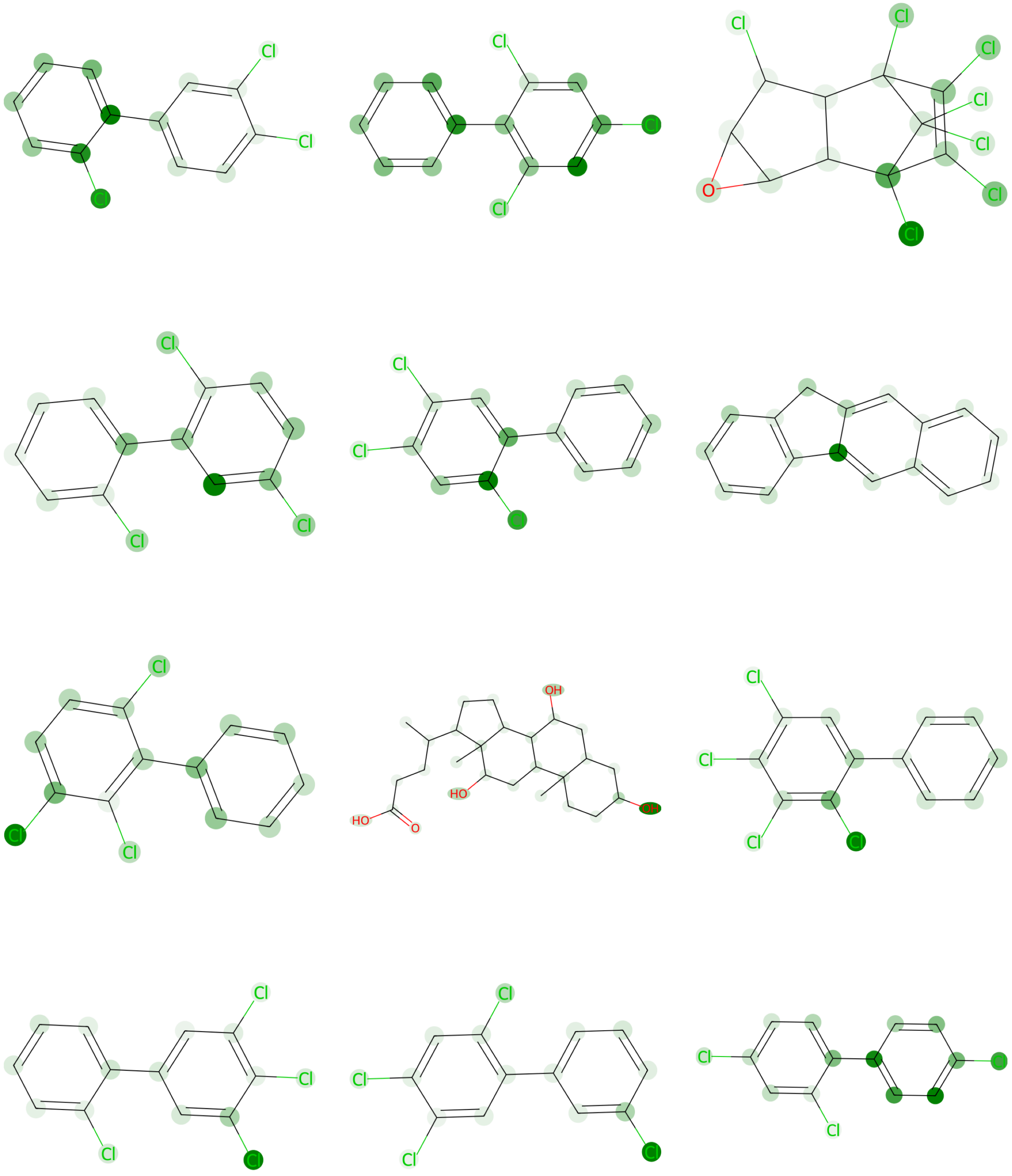

Fig. S 11 Visualizations of the molecular neighborhood of cluster two, obtained through k-means (k=4) clustering on the 2d PCA-reduced embeddings (Fig. S4) and selecting the nearest neighbors based on 2d PCA distance. The corresponding colorbar can be seen in Fig. S5, top.

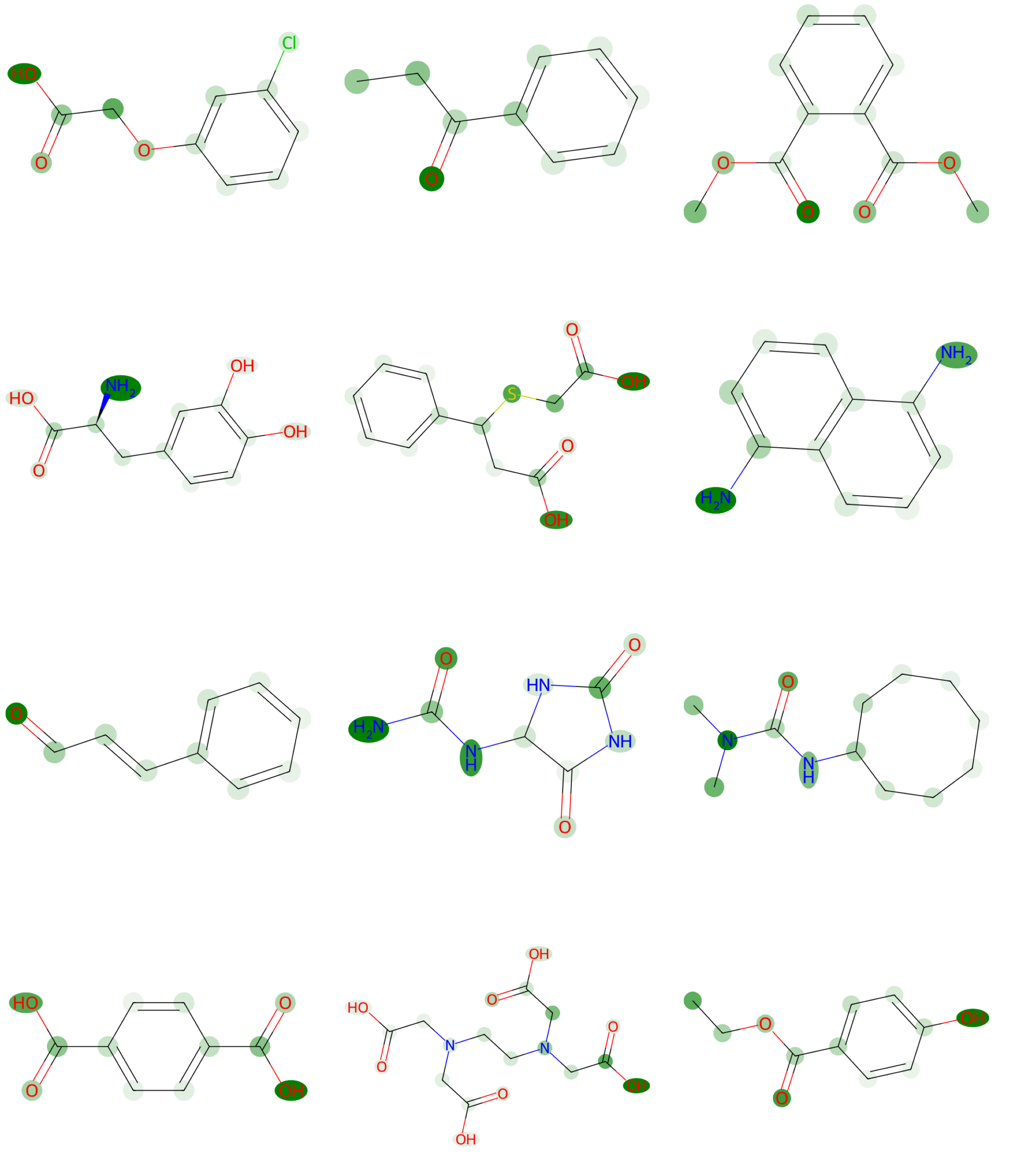

Fig. S 12 Visualizations of the molecular neighborhood of cluster three, obtained through k-means (k=4) clustering on the 2d PCA-reduced embeddings (Fig. S4) and selecting the nearest neighbors based on 2d PCA distance. The corresponding colorbar can be seen in Fig. S5, top.

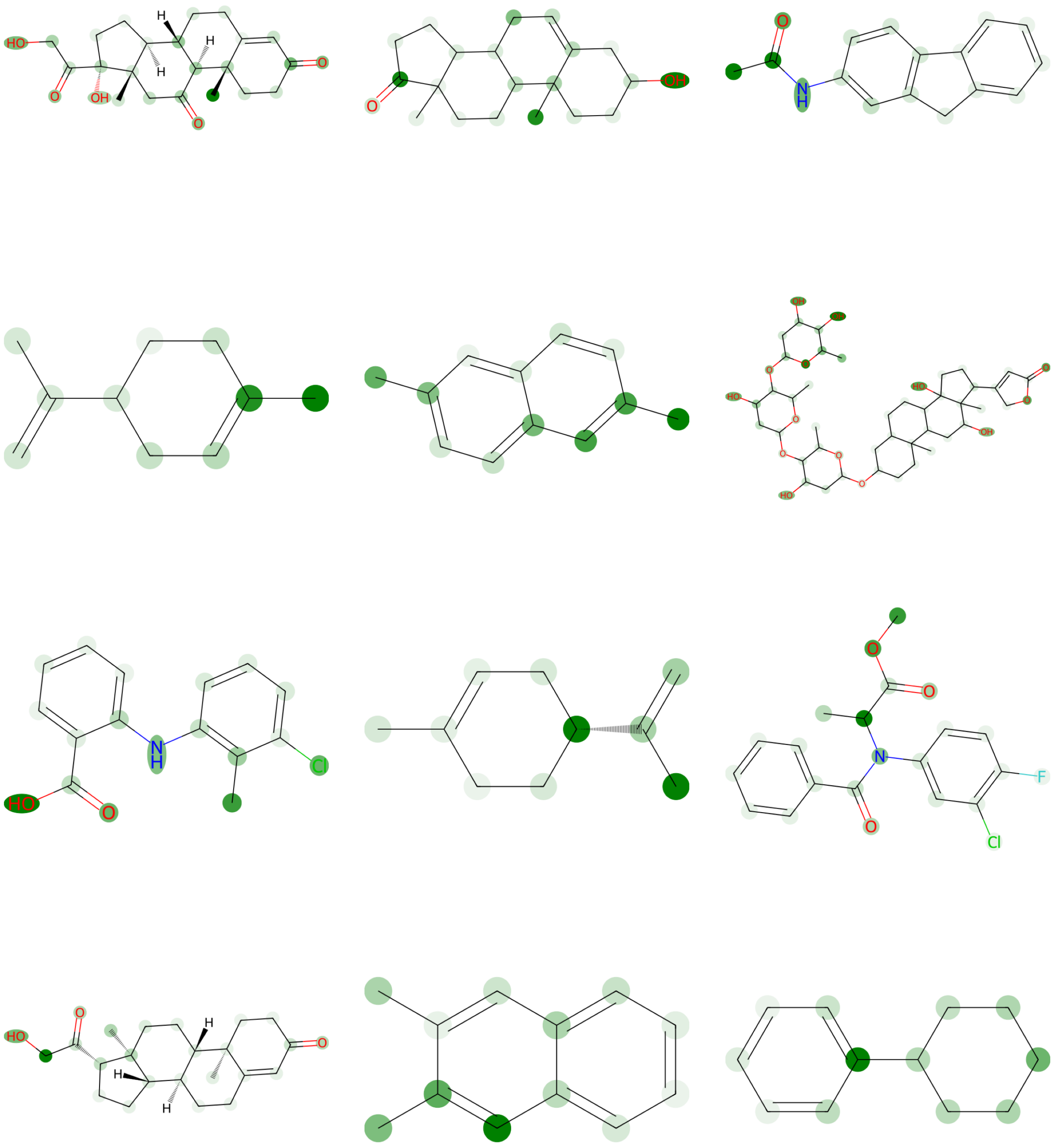

Fig. S 13 Visualizations of the molecular neighborhood of cluster four, obtained through k-means (k=4) clustering on the 2d PCA-reduced embeddings (Fig. S4) and selecting the nearest neighbors based on 2d PCA distance. The corresponding colorbar can be seen in Fig. S5, top.

8 Comparison of ECFP attributions from 512-bit and 2048-bit fingerprints

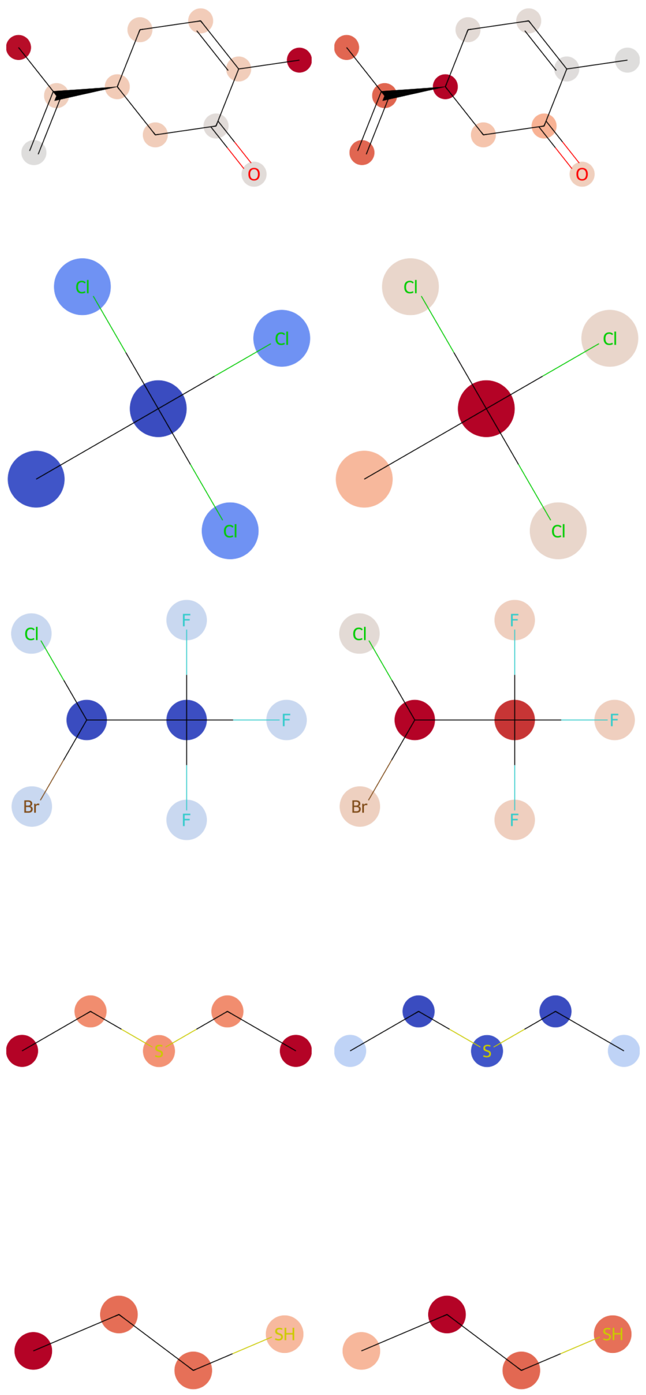

Fig. S 14 Visualizations of ECFP attributions comparing the impact of ECFP fingerprint bit size, specifically comparing ecfp-lin-scaled (512 bits, left) and ecfp2k-lin-scaled (2048 bits, right). The molecules from the same cluster neighborhoods as Fig S4 is shown. The corresponding colorbar can be seen in Fig. S5, bottom.

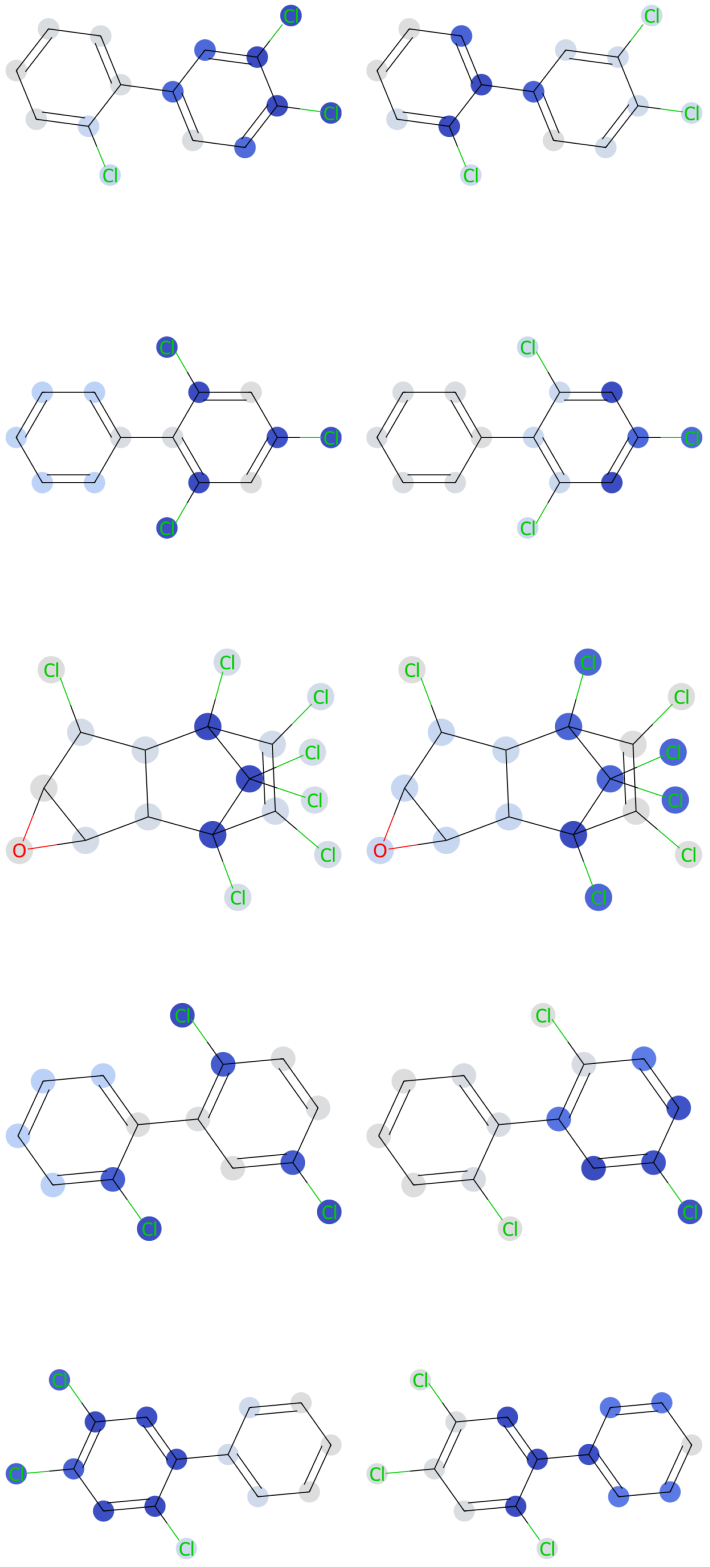

Fig. S 15 Visualizations of ECFP attributions comparing the impact of ECFP fingerprint bit size, specifically comparing ecfp-lin-scaled (512 bits, left) and ecfp2k-lin-scaled (2048 bits, right). The molecules from the same cluster neighborhoods as Fig S4 is shown. The corresponding colorbar can be seen in Fig. S5, bottom.

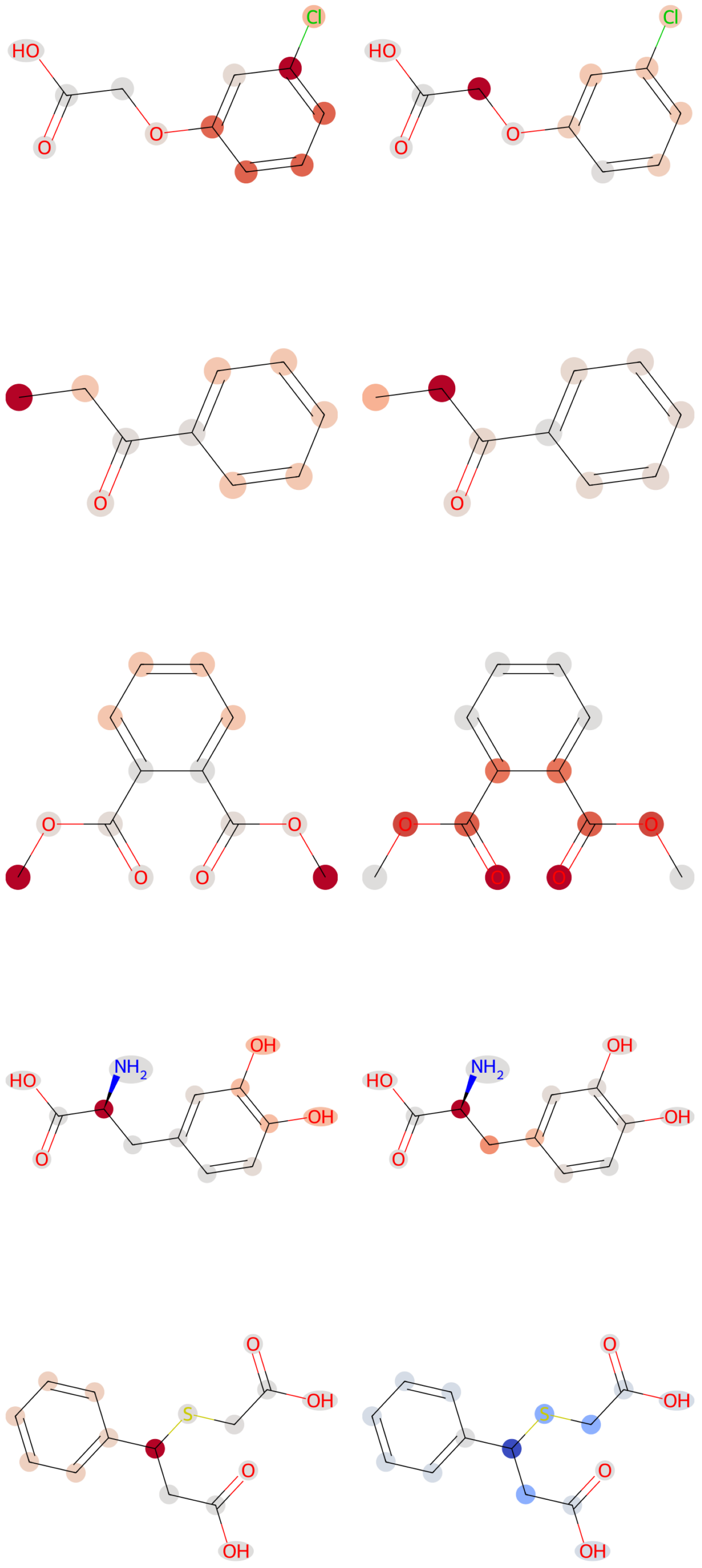

Fig. S 16 Visualizations of ECFP attributions comparing the impact of ECFP fingerprint bit size, specifically comparing ecfp-lin-scaled (512 bits, left) and ecfp2k-lin-scaled (2048 bits, right). The molecules from the same cluster neighborhoods as Fig. S4 is shown. The corresponding colorbar can be seen in Fig. S5, bottom.

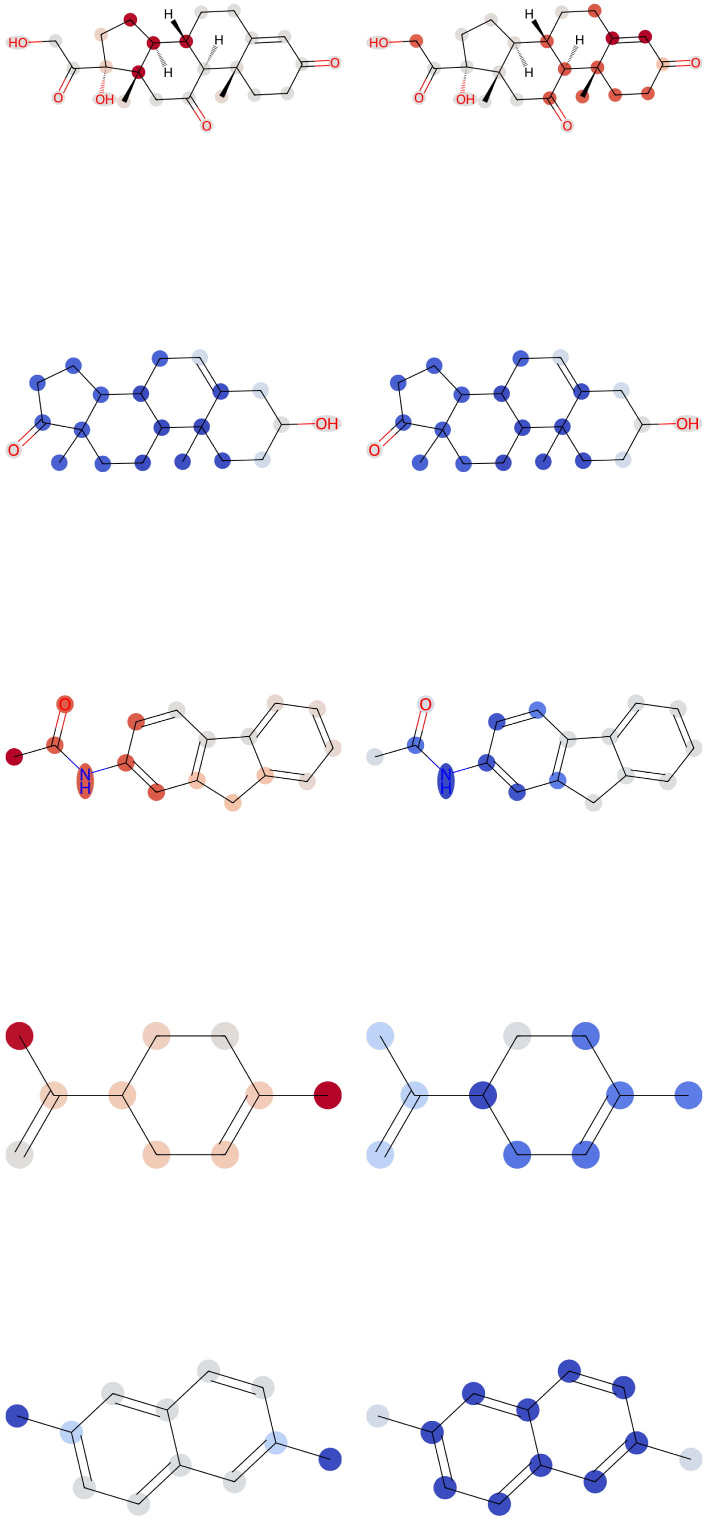

Fig. S 17 Visualizations of ECFP attributions comparing the impact of ECFP fingerprint bit size, specifically comparing ecfp-lin-scaled (512 bits, left) and ecfp2k-lin-scaled (2048 bits, right). The molecules from the same cluster neighborhoods as Fig S4 is shown. The corresponding colorbar can be seen in Fig. S5, bottom.

9 Attribution comparison for the "random" test set

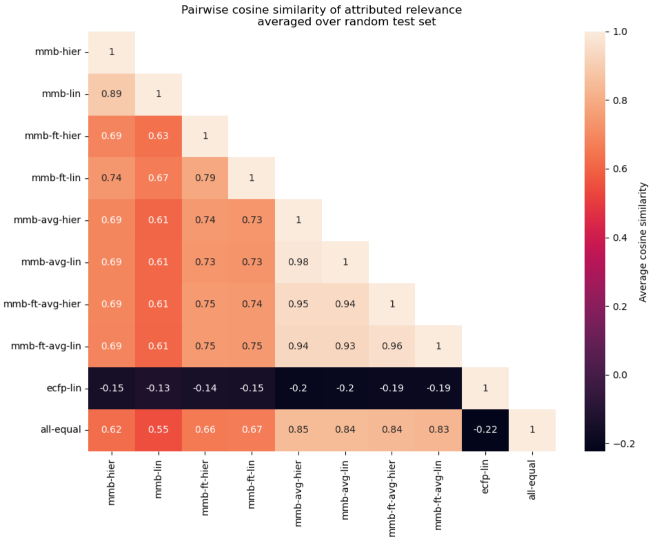

Fig. S 18 Quantitative comparison of attributed atom relevance for a random test set. For each model pair, the cosine similarity of attributed relevance is calculated as the average over all molecules in the test set. The first four MMB models use the <R> token and attribution method developed in this work, while the middle 4 MMB models use the <avg> pooling approach in combination with SHAP to obtain attributions. For both models, finetuning (-ft-) and regression head variants (lin or hier) are compared. ECFP attributions are extracted by attributing regression weights to all constituent atoms and aggregating per-atom contributions. The last entry shows uniform relevance, a baseline where every atom has the same normalized attributed importance towards the prediction.

10 Attribution of negative features

As discussed in the main text, we find features with negative contributions cannot be attributed towards the input due to the all-zero gradients of the attention heads. Specifically, we obtain all-zero $\nabla A_h^{-,l}$ matrices in all 6 layers $l \in L$ for all 8 attention heads $h \in 0, 1, .., 7$ for the finetuned <R> MMB model with a linear regression head (mmb-ft-lin) when we mask all features of the latent vector that have an overall positive sign (see Fig. S19 and Fig. S20). However, features with negative contributions constitute a significant part of the prediction. We emphasize that the regression prediction itself is unaffected and accurate, with the positive and negative contributions adding up to the overall prediction, which is independent of the explainability approach. Since most than half of the features have a negative contribution on the log-scale solubility, we find the majority of features are ignored, and only those features which contribute positively are attributed and visualized. We further highlight that this limitation is not directly due to the positive importance assumption $(\cdot)^+$ in Eq. 2, since the gradient $\nabla \mathbf{A}_h^l$ is already all-zero for features with negative contributions before the update rule enforces positive importance.

This implies that in this context, *only* the positive part of the prediction is attributed and visualized, misrepresenting the model's operation by disregarding all negative contributions. The same holds for interactions between multiple features, such as the presence of one but not another functional group, which can be expected to involve opposing signs and are thus misrepresented by the explainability technique in this context. Whether this finding has relevance beyond the attribution method and chemical language model analyzed in this work goes beyond the scope of this study. However, our findings suggest an important direction for further research to investigate whether this limitation affects other gradient-based attribution methods and modalities beyond molecular strings.

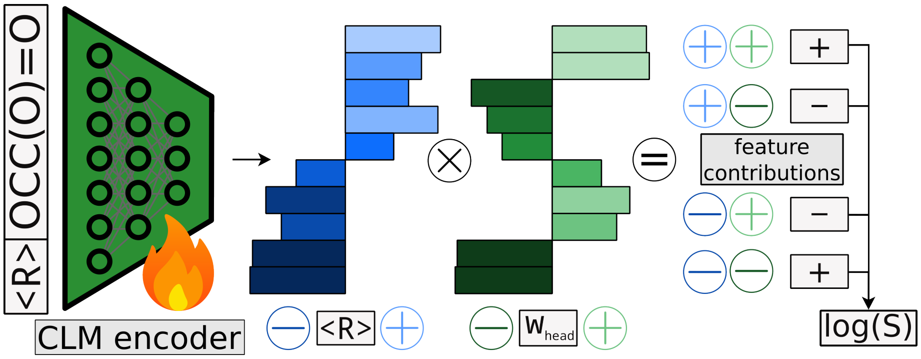

Fig. S 19 Schematic illustration of the features divided into four types of contributions, which either positively or negatively change the overall prediction. The encoder of the chemical language model takes the tokenized SMILES string as input and produces a latent feature vector leveraging the prepended <R> token. The sum of all feature contributions, obtained as the product between the activation vector (blue) and the weights vector of the linear regression head (green), is the scalar $\log(S)$ prediction of the model. The contribution from each feature towards the prediction is separated into an overall positive contribution (obtained from features with the same sign in the activation and regression vector) and an overall negative contribution (from opposite signs). All four kinds of features significantly contribute to the prediction, but our attribution technique only attributes relevance from positive features and thus ignores significant parts of the model.

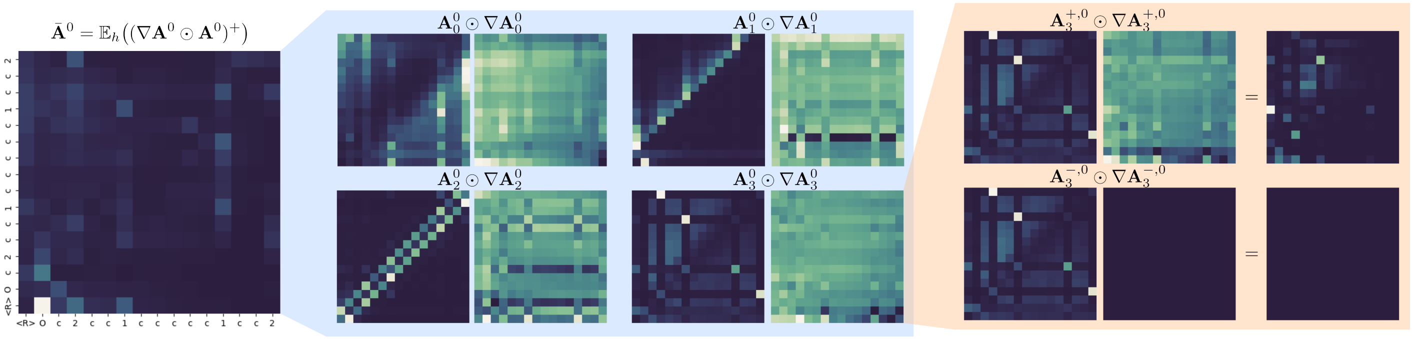

Fig. S 20 Visualization of attributed relevance $\bar{A}$ of one molecule of the first layer (left), illustrating how individual attention heads are aggregated. $\bar{A}^l$ (left) is calculated as the element-wise matrix product of each attention head and its gradient ($\mathbf{A}_h^l \odot \nabla \mathbf{A}_h^l$), averaged over all 8 attention heads in a layer. Activation heatmaps and their gradients of the first four attention heads of the first layer are visualized (blue).
The heatmaps on the right (orange) visualise the activation & gradient of an individual attention head (layer 0, head 3) when features with positive ($\mathbf{A}_3^{+,0}$, top) or negative ($\mathbf{A}_3^{-,0}$, bottom) contributions are attributed separately by masking all features with opposite contributions. The gradient of the negative features $\nabla \mathbf{A}_3^{-,0}$ is all-zero, thus no relevance is attributed for all features with an overall negative contribution despite their contribution towards the prediction.
